# Potential application of mesenchymal stem cells and their exosomes in lung injury: an emerging therapeutic option for COVID-19 patients

**DOI:** 10.1186/s13287-020-01963-6

**Published:** 2020-10-15

**Authors:** Sara Al-Khawaga, Essam M. Abdelalim

**Affiliations:** 1 Dermatology Department, Hamad Medical Corporation, Doha, Qatar; 2 Weill Cornell Medicine-Qatar, Qatar Foundation, Doha, Qatar; 3 Diabetes Research Center, Qatar Biomedical Research Institute (QBRI), Hamad Bin Khalifa University (HBKU), Qatar Foundation (QF), PO Box 34110, Doha, Qatar; 4 College of Health and Life Sciences, Hamad Bin Khalifa University (HBKU), Qatar Foundation, Education City, Doha, Qatar

**Keywords:** Stem cells, MSCs, SARS-CoV-2, ARDS, Exosome, Treatment, Clinical trials, Pneumonia

## Abstract

The COVID-19 pandemic has negatively impacted the global public health and the international economy; therefore, there is an urgent need for an effective therapy to treat COVID-19 patients. Mesenchymal stem cells (MSCs) have been proposed as an emerging therapeutic option for the SARS-CoV-2 infection. Recently, numerous clinical trials have been registered to examine the safety and efficacy of different types of MSCs and their exosomes for treating COVID-19 patients, with less published data on the mechanism of action. Although there is no approved effective therapy for COVID-19 as of yet, MSC therapies showed an improvement in the treatment of some COVID-19 patients. MSC’s therapeutic effect is displayed in their ability to reduce the cytokine storm, enhance alveolar fluid clearance, and promote epithelial and endothelial recovery; however, the safest and most effective route of MSC delivery remains unclear. The use of poorly characterized MSC products remains one of the most significant drawbacks of MSC-based therapy, which could theoretically promote the risk for thromboembolism. Optimizing the clinical-grade production of MSCs and establishing a consensus on registered clinical trials based on cell-product characterization and mode of delivery would aid in laying the foundation for a safe and effective therapy in COVID-19. In this review, we shed light on the mechanistic view of MSC therapeutic role based on preclinical and clinical studies on acute lung injury and ARDS; therefore, offering a unique correlation and applicability in COVID-19 patients. We further highlight the challenges and opportunities in the use of MSC-based therapy.

## Background

In December 2019, the severe acute respiratory syndrome coronavirus 2 (SARS-CoV-2) has been identified as the cause of a respiratory illness coronavirus disease 2019 (COVID-19) [1]. The most common treatment for COVID-19 patients remains to be supportive care. Despite the emerging therapeutic agents have been assessed for the treatment of COVID-19, none has yet been shown to be efficacious [2, 3]. To date, no dedicated therapeutic agent has been implemented yet, nor a vaccination strategy that has been confirmed to prevent COVID-19. The case fatality rate (CFR) has been estimated by the WHO to range from 0.3 to 1%, higher than that of influenza A [4].

Immune-mediated lung injury and acute respiratory distress syndrome (ARDS) are associated with poor prognosis in COVID-19 patients [5]. Symptoms of COVID-19 usually range from mild upper respiratory tract symptoms to progressive life-threatening viral pneumonia and progressive hypoxemia requiring mechanical ventilatory support. The leading cause of mortality in COVID-19 patients is hypoxemic respiratory failure most frequently resulting in ARDS, characterized by diffuse lung damage with edema, hemorrhage, and intra-alveolar fibrin deposition [6, 7]. More interestingly, laboratory findings indicate a hyperactivated nature of the immune system, specifically high levels of circulating CD4^+^ and CD8^+^ lymphocytes. Looking at the hyperactive immune response detected in COVID-19 patients, several potential treatments relating to key immunoregulators have been proposed. Another important factor influencing the prognosis of COVID-19 patients is having a state of hyperinflammation, where several immunosuppression modalities have provided a tool to decrease the mortality in patients with severe condition [8]. Understanding the pathogenesis of SARS-CoV-2 in association with the host immune response will help elucidate some key targeted treatment options. Repurposing of previously approved medications, such as the anti-malarial drug hydroxychloroquine, anti-rheumatic drugs, such as tocilizumab (interleukin [IL]-6 receptor inhibitor), baricitinib (Janus kinase [JAK] inhibitor), and anakinra (IL-1 receptor antagonist), have been employed to treat COVID-19, largely attributed to their known pharmacokinetic and safety profiles [9].

Mesenchymal stromal/stem cells (MSCs) offer a promising emerging therapeutic approach toward modifying the adverse effects of the infection in SARS-CoV-2 patients. This therapy has been found to decrease the cytokine storm and exert anti-inflammatory, immunomodulatory, and regenerative functions by altering the expression of pro-inflammatory cytokines, and aid in repairing the damaged tissues in COVID-19 patients. Several clinical trials have already provided a proof of concept showing that intravenous (IV) infusion of MSCs is a safe option and could lead to clinical and immunological improvement in some patients with severe COVID-19 pneumonia [10]. Such findings support employing phase 2 randomized controlled trial, where other randomized trials with a control arm consisting of standard treatment, will help to elucidate the mechanistic potential of MSC-based therapeutic strategy. This review summarizes the immunopathogenesis of the SARS-CoV-2 and the therapeutic potentials of MSCs for treating lung injuries associated with COVID-19. Furthermore, we highlight the current clinical trials using MSCs for treating COVID-19 patients and discuss limitations of the existing MSC-based treatment strategies.

## Pathogenesis of SARS-COV-2

The lung alveoli are lined with the alveolar epithelium consisting of a monolayer of alveolar type I (AT1) cells and alveolar type II (AT2) cells. Under normal condition, the AT2 cells secrete surfactant covering all the lining epithelium to facilitate alveolus expansion. AT1 and AT2 are tightly connected with tight junctions, which control the transfer of ions and fluid across the epithelium. The endothelial cells of the blood capillaries are connected by intercellular junctions and control the influx of inflammatory cells and fluid into the interstitial space between the aveoli. Initially, the spike glycoprotein (S protein) expressed on viral envelopes binds to the angiotensin-converting enzyme 2 (ACE2) receptor [11], a very similar structure to that of SARS; however, with a 10–20 times much higher binding affinity when compared to the SARS S protein [12]. This binding capability partially explains the high transmission of SARS-CoV-2 [12]. The main target cells for SARS-CoV-2 infection are AT2 cells and resident alveolar macrophages, because they are expressing ACE2. SARS-CoV-2 utilizes ACE2 for entry and the serine protease TMPRSS2, which is also expressed by the alveolar cells, for S protein priming [13]. This activation induces chemokine and cytokine secretion that recruits inflammatory and immune cells into the infected alveoli, followed by other waves of cytokine release. Activated macrophages have a significant role in hemophagocytic lymphohistiocytosis (HLH)-like cytokine storm during COVID-19 [14]. Secondary HLH could be precipitated by a genetic defect in cytolytic pathways or observed in during infection, malignancy, and rheumatic disease. HLH is characterized by a predominance of inflammatory cytokines and expansion of tissue macrophages displaying hemophagocytic activity [15]. Cytopenias, a state of elevated inflammatory cytokines or hypercytokinaemia, unremitting fever, elevated ferritin level, and multi-organ damage, are among the key characteristics of HLH seen in seriously ill COVID-19 patients [8]. Type I interferons (IFN) and natural killer (NK) cells result in cytolytic immune responses, following a successful recognition of pathogen-associated molecular pattern. This serves as a first line of defense against SARS-CoV-2 infection through the innate immune system. Activated cytotoxic T cells and B cells are key players of the adaptive immunity helping with viral clearance via destruction of virus-infected cells and antibody production, respectively. However, when the anti-viral immune response remains active, an aberrant and uncontrolled production of inflammatory cytokines occurs, causing what is known as the “cytokine storm”, leading to damage in the pulmonary tissue [16, 17].

Severely ill COVID-19 patients, especially the ones with pneumonia, show disproportionate immune profile, with considerably lower lymphocyte counts (lymphocytopenia) and increased concentrations of inflammatory cytokines. Among the significant inflammatory interleukins (ILs) are IL2, IL-6, IL-7, IL-10 (Th2), IL-1β and IFNγ (Th1), and tumor necrosis factor (TNF) [6]. Furthermore, in patients with severe symptoms, an elevation in granulocyte-colony stimulating factor (G-CSF), IFNγ-induced protein-10 (IP-10), macrophage inflammatory protein 1α (MIP-1α/CCL3), and macrophage chemoattractant protein-1 (MPC-1/CCL2) are noticed [18]. A recent study has performed a screen for 48 cytokines in 53 COVID-19 patients with moderate and severe symptoms recorded a dramatic increase of 14 cytokines in COVID-19 patients in comparison to healthy individuals [19]. Of those cytokines, the increased hepatocyte growth factor (HGF), MCP3, IP-10, monokine induced gamma interferon (MIG), and MIP1α are associated with the severity of the symptoms [19]. Key cells in the adaptive immunity, such as CD4^+^ T cells, CD8^+^ T cells, and NK cells are also decreased in severely ill patients [5]. On the other hand, an elevation of CD14^+^ CD16^+^ monocytes, IL-17-producing CCR4^+^ CCR6^+^ CD4^+^ (T-helper 17/Th17) cells, perforin and granulysin-expressing cytotoxic T cells are reported. These constitute the pro-inflammatory subsets of T cells responsible for the severe immune injury in the lungs [5].

**Fig. 1:**
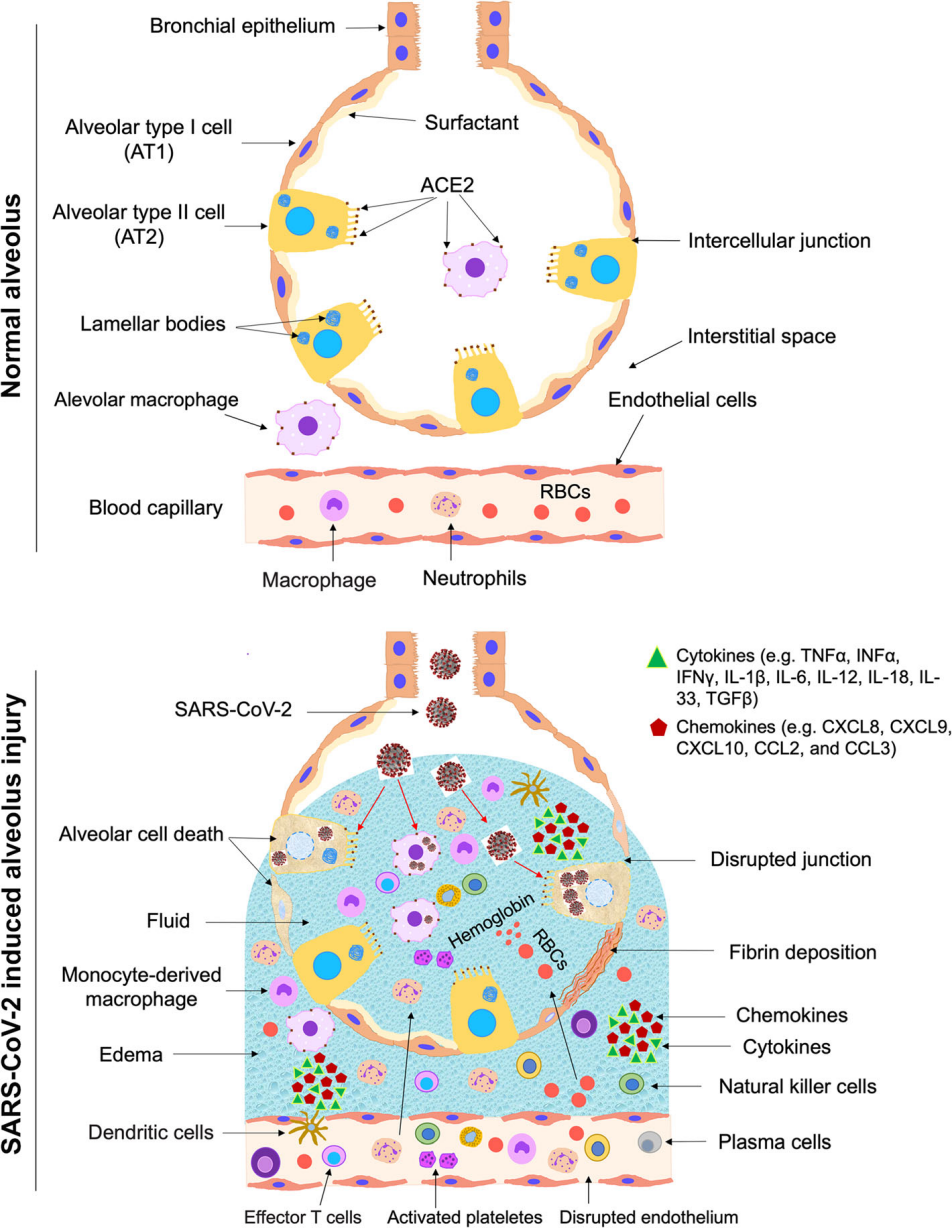
Immunopathogenesis of the SARS-CoV-2. The alveolar epithelium consists of a monolayer of alveolar type I (AT1) cells and alveolar type II (AT2) cells. Under normal condition, the AT2 cells secrete surfactant covering all the lining epithelium to facilitate alveolus expansion. AT1 and AT2 are tightly connected with tight junctions, which control the transfer of ions and fluid across the epithelium. The endothelial cells of the blood capillaries are connected by intercellular junctions and control the influx of inflammatory cells and fluid into the interstitial space. SARS-CoV-2 infects AT2 cells and resident alveolar macrophages that express ACE2. This activation induces chemokine secretion that recruits inflammatory and immune cells into the infected alveoli. The increased inflammatory cells in the lung lead to secretion of large amounts of pro-inflammatory cytokines “cytokine storm” that lead to damages in the lung. The migrated neutrophils and monocytes release toxic mediators, causing endothelial and epithelial injuries. The intercellular junctions are disrupted leading to formation of gaps between the alveolar cells as well as between the endothelial cells, resulting in an increase in the permeability of the epithelial and endothelial cells. The increase in the permeability facilitates the migration of inflammatory cells and allows the influx of RBCs and fluid from the blood capillary. Large volume of fluid (alveolar edema) fills the airspace leading to a difficulty in the breathing. Also, the inflammatory reactions may lead to alveolar cell death, fibrin deposition, and hyaline membrane formation

**Fig. 2:**
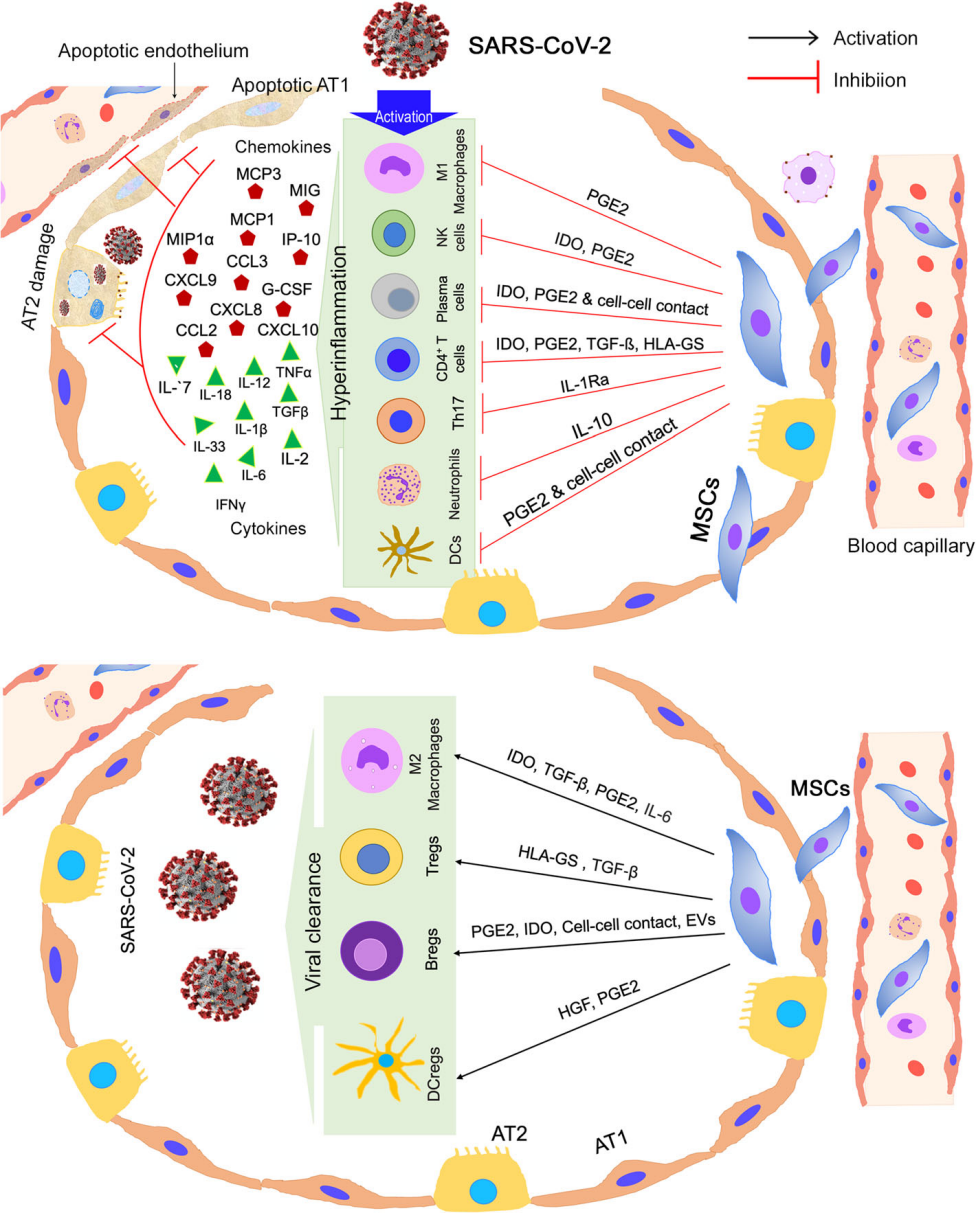
Anticipated immunomodulatory actions of MSCs in the lung infected with SARS-CoV-2. MSCs perform immunomodulatory functions by multiple ways, including cell-cell contact, paracrine factor secretion, and extracellular vesicles (EVs). Upper panel shows the inhibitory effect of MSCs on immune cells, which are highly activated by the viral infection and secrete chemokines and cytokines in response to the infection. These chemokines and cytokines increase lung inflammation and cause epithelial and endothelial damage. Lower panel show the stimulatory effect of MSCs on other immune cells, which are crucial for SARS-CoV-2 clearance. AT1, alveolar type I epithelial cells; AT2, alveolar type II epithelial cells; NK cells, natural killer cells; Th17, T helper 17 cells; DCs, dendritic cells; DCregs, regulatory dendritic cells; Tregs, regulatory T cells; Bregs, regulatory B cells

**Fig. 3:**
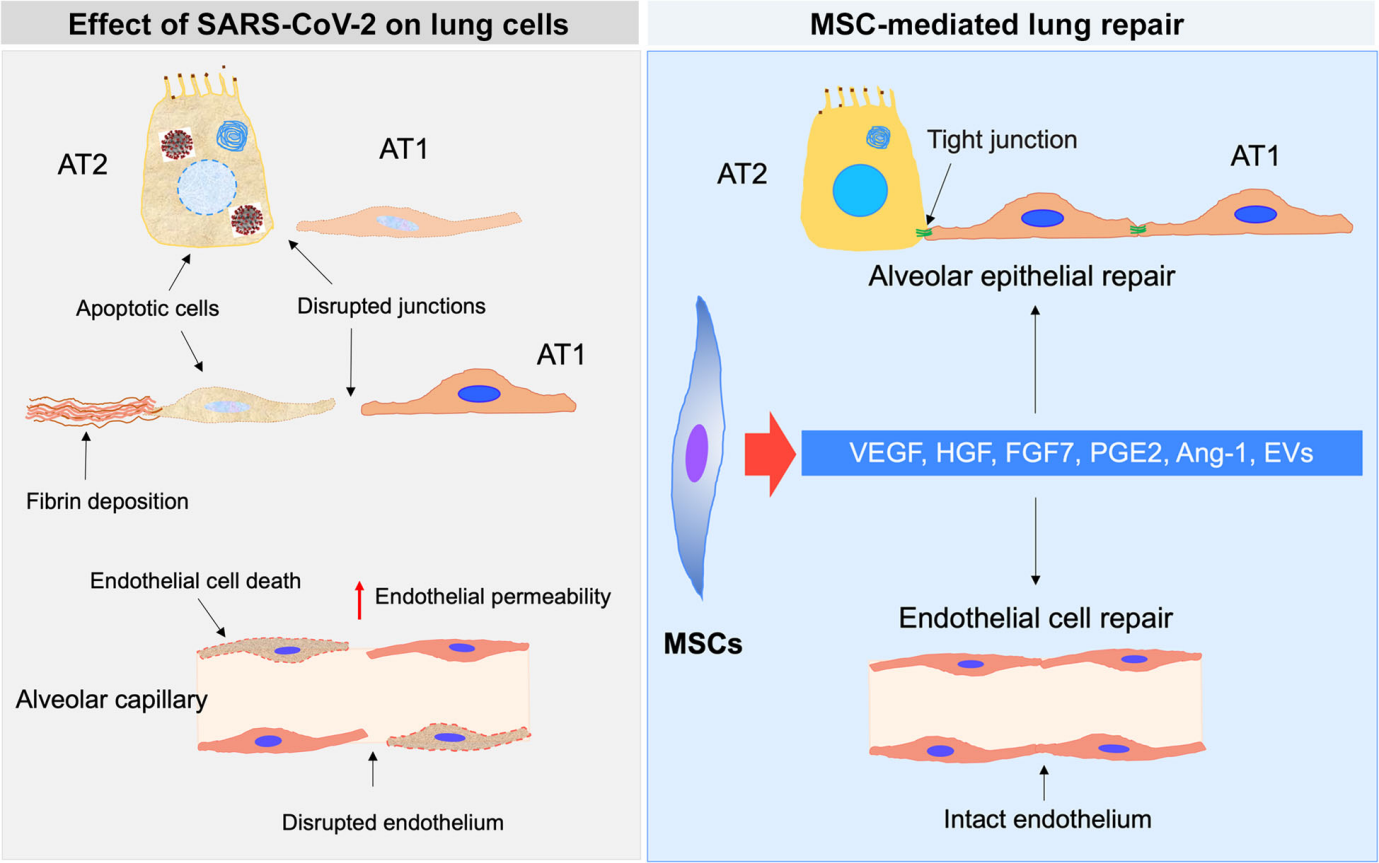
Anticipated effect of MSCs on lung cells in SARS-CoV-2-induced lung injury. High levels of pro-inflammatory cytokines (cytokine storm) associated with SARS-CoV-2 infection lead to lung cell damage and an increase in the permeability of pulmonary capillaries. The affected cells include alveolar type I epithelial cells (AT1), alveolar type II epithelial cells (AT2), and endothelial cells. MSCs secrete several paracrine factors and extracellular vesicles (EVs), which have anti-apoptotic functions. This effect enhances cell survival and improves lung functions. Ang-1, Angiopoietin 1; HGF, hepatocyte growth factor; FGF7, fibroblast growth factor-7; VEGF, vascular endothelial growth factor

Among the histological profiles of COVID-19 are the significant alterations in the morphology of the endothelial cells, which also express ACE2. These changes include damage of the intercellular junctions, a loss of attachment to the basement membrane, and cell swelling [20] (Fig. 1). The migrated neutrophils and monocyte-derived macrophages release toxic mediators, causing endothelial and epithelial injuries (Figs. 1 and 2). The intercellular junctions are disrupted leading to formation of spaces between the alveolar cells as well as between the endothelial cells, resulting in an increase in the permeability of the epithelial and endothelial cells (Figs. 1, 2, and 3). The increase in the permeability facilitates the migration of inflammatory cells and allows the influx of RBCs and fluid from the blood capillary. Large volume of fluid (alveolar edema) fills the airspace leading to a difficulty in the breathing. Also, the inflammatory reactions may lead to alveolar cell death, fibrin deposition, and hyaline membrane formation. These findings support an important role of endothelial cells in the vascular phase of COVID-19. Furthermore, pulmonary intussusceptive angiogenesis and other pulmonary vascular lesions have been observed in autopsy specimen of COVID-19 patients [20].

Severe respiratory illness could be a major symptom of SARS-CoV-2 infection, because the ACE2 receptor is expressed in the lung AT2 cells, alveolar macrophage, and capillary endothelial cells [11] (Fig. 1). The expression of the ACE2 has been detected in other tissues, such as the cardiovascular, hepatic, renal, pancreatic, and the gastrointestinal tissues. This expression profile partially explains why some infected patients not only develop ARDS, but also develop other complications, such as myocardial injury (MI), arrhythmia, acute kidney injury (AKI), shock, multi organ failure, diabetes, and ultimately death [21].

IL-6 has an essential part in inflammatory cytokine storm in COVID-19. IL-6-producing CD14^+^ CD16^+^ inflammatory monocytes are significantly high [22]; therefore, the rationale for using tocilizumab has been used in COVID-19 patients. Tocilizumab, which is a recombinant humanized monoclonal antibody against the IL-6 receptor, is likely to induce its antagonistic effect on IL-6-producing monocytes following activated Th1 cells in the lung. Tocilizumab is a first drug for the treatment of cytokine storm in COVID-19, especially in patients with multiple comorbidities. Despite the numerous ongoing trials assessing the safety and efficacy of tocilizumab in COVID-19 patients, IL-6 play a role in controlling the lung inflammation and is important for the clearance of viruses [23]. Therefore, inhibiting IL-6 raises the possibility of impaired viral clearance or exacerbation of lung inflammation [9].

Interestingly, an abnormal coagulation profile has been shown in COVID-19 patients during the late stage of the disease; specifically, increased concentrations of D-dimer and other fibrin degradation products are mainly associated with poor prognosis [24]. The HScore is a recommended evaluation as well as prognostic tool used in patients with secondary HLH at high risk of hyperinflammation. The score combines both critical laboratory as well as clinical parameters, assessing for an underlying of immunosuppression and cytopenias, measuring serum aspartate aminotransferase (AST), triglycerides, fibrinogen, ferritin, body temperature, organomegaly, and hemophagocytosis on bone marrow aspirate [8]. The HScores generate a probability for the presence of secondary HLH; a score more than 169 is 93% sensitive and 86% specific for HLH [8].

Finally, since the anti-viral immunity is needed to recover from COVID-19, the use of immunosuppressants on these patients should be used with caution. One strategy to avoid the inhibition of anti-viral immunity is to use targeted instead of broad immunosuppressive medications. Unfortunately, we still lack consensus on the optimal timing of treatment administration to decrease the harmful effects of immunosuppression, as well as the routes of their administration.

## Mesenchymal stem cells (MSCs): characteristics and types

MSCs are a heterogeneous cell population propagating in vitro as plastic-adherent cells, have fibroblast-like morphology, and form colonies in vitro [25]. The International Society for Cellular Therapy (ISCT) defining criteria for MSCs is that they adhere to plastic, express the surface markers CD90, CD73, and CD105, are negative for the hematopoietic markers CD14, CD34, CD45, CD19, and HLA-DR, and should express a multilineage differentiation capability into adipogenic, osteogenic, and chondrogenic lineages [26].

Bone marrow MSCs (BM-MSCs) are considered the most widely used and investigated type of MSCs, which was first isolated from the bone marrow by Friedenstein and colleagues in 1974 [27]. Later, MSCs were identified and successfully produced from other sources, such as the perivasculature [28], adipose [29], dental pulp [30], muscle [31], dermis [31], and fetal tissue [32]. The abundance of adipose-derived stem cells (ASCs), their ease of isolation using a minimally invasive procedure [33], and their expansion as well as their differentiation ability into multiple lineages make ASCs a promising less-invasive alternative to BM-MSCs for therapeutic applications [34, 35]. The most commonly used adult sources for human MSCs are bone marrow [36] and the adipose tissue stromal vascular fraction [29, 34, 35]. The highly harvestable bone marrow or unwanted/waste product of adipose sources forms the foundation for most of the data in the field of MSC-based therapeutics. The umbilical cord (UC) tissue [37] and the placenta [38, 39] and their associated tissue Wharton jelly (WJ), and amniotic fluid (AF), are among the other young “adult” tissues, that are also considered good sources of human MSCs, where they are normally discarded after birth.

MSCs are generally recognized as immune evasive making them safe when used in allogeneic settings [40]. Allogeneic MSCs are able to bypass the immune system due to low expression of the major histocompatibility complex-1 (MHC-I) and -II proteins. MSCs are often referred to as being “immunoprivileged” due to lack of the T cell costimulatory molecules, CD80 and CD86 [41]. Previous studies reported that fetal MSCs, adult BM-MSCs, and ASCs express HLA-I and do not express HLA-II [42–46]; however, these MSCs start to express HLA-II after stimulation with IFN-γ [44, 45, 47, 48]. A recent study demonstrated that iPSC-derived MSCs do not express HLA-II and costimulatory molecules [49]. Interestingly, induced pluripotent stem cell (iPSC)-derived MSCs express a very low level of HLA-II in comparison to MSCs derived from fetuses and adult sources after their stimulation with interferon-γ (IFN-γ) [49]. These findings present iPSC-MSCs as an efficient source for allogenic transplantation without the risk of immune rejection due to the lower immunogenicity compared to adult MSCs.

## Therapeutic potentials of MSCs

MSCs have been extensively studied over the past ~ 30 years for their wide clinical applications and regenerative capacity. MSCs have made their way over the past 25 years into now over 950 registered clinical trials listed with the FDA, exhibiting an excellent safety profile. With over a 10,000 patients treated with MSCs in a controlled clinical setting, and upon successful completion of phase 1 or phase 2 trials, several tens of MSCs-based studies have advanced to phase 3 clinical trials (www.clinicaltrials.gov).

A fundamental clinical decision remains to choose among whether to use autologous vs allogeneic sources of MSCs, where both have displayed successful production of large numbers of MSCs [50, 51]. MSC replacement in the large numbers is needed to treat significant tissue injury, a process that further requires orchestrated steps involving successful engraftment and cell differentiation [52]. A target dose of 100–150 million MSCs can be obtained from cell culturing and expansion of 25 ml of BM-MSC aspirate. In about 3 weeks duration, a volume of about 0.4–0.5 ml of packed cells can be generated [53]. The MSC isolation from different tissues, such as BM and adipose tissue and their re-implantation at other sites highlight their ability to repair tissues in vivo. However, this process clearly diminishes in aging population compared to younger adults [54]. In addition, MSCs could be generated in vitro in large number from human pluripotent stem cells (hPSCs) [55, 56], which showed a lower immunogenicity in comparison to adult sources [49].

MSCs have been extensively examined for their therapeutic capacity in regenerative medicine, because of their ability to home to sites of inflammation and damaged tissue, ultimately serving as a source of growth and trophic factors and regenerative molecules. The potential therapeutic effect of MSCs is based on their low immunogenicity, their immunomodulatory characteristics, and their ability to secrete growth factors, as well as anti-microbial peptides [57]. MSCs administered systemically tend to migrate to the injury region to promote functional recovery [58]. MSCs can also extravasate from the blood vessels, just like immune cells, via the expression of cell surface adhesion molecules. Migration of MSCs occur in response to chemokines binding to cognate receptors present on their cell surface [59] and result in the stimulation of matrix metalloproteinases degrading the basal membrane and allowing subsequent extravasation [60]. By displaying a coordinated rolling, MSCs contact the endothelial cells in a P-selectin- and vascular cell-adhesion molecule 1 (VCAM1)-dependent manner [61]. Guided by chemotactic signals, MSCs migrate through the interstitium to the injured area. An increase in the MSC migration capacity toward chemokines is achieved via the upregulation of their receptors, CCR2, CCR3, and CCR4. Also, interleukin (IL)-8, an inflammatory chemokine, may induce migration of MSCs to injured areas [62, 63].

### Immunoregulatory functions of MSCs

One of the major therapeutic characteristics of MSCs is their immunomodulatory role, including a network of cytokines and cell-cell interactions. Interestingly, MSCs only exert its immunoregulatory capacity after receiving the activation signals from the inflammatory milieu; therefore, MSC’s immunoregulatory capacity is not constitutive, rather is driven by “licensing” process [64]. Previous studies showed that the macrophages play an essential role during wound healing; thus, they have emerged as key candidate targets in therapeutic tissue regeneration approaches [64, 65]. Macrophages exhibit functional repolarization as tissue repair progresses, shifting from the pro-inflammatory or M1-phenotype to an anti-inflammatory or M2-phenotype. M1 macrophages secrete high levels of pro-inflammatory cytokines, while M2 macrophages secrete lower levels of pro-inflammatory cytokines, exhibit tissue repair, and enhance the resolution of inflammation [66]. Imbalance between M1- and M2- activities can lead to continuous inflammation and hinders the normal repair process, both contributing to impaired tissue repair [67]. MSCs enhance tissue repair and regeneration by modulating the immune response, acting as sensors and switchers of inflammation, rather than by replacing damaged cells. This is largely attributed to the secretion of growth factors; among the immunoregulatory factors are prostaglandin E2 (PGE2) and IL-6 that help in transitioning macrophages toward M2 phenotype [68, 69] (Fig. 2). Further, the classical pro-inflammatory cytokines produced at the acute stage of inflammation, such as IFN-γ, TNF-α, or IL-1β enhances the paracrine effects of MSCs exerted on macrophages [70, 71].

In order to stimulate the MSC immunosuppressive effect, threshold levels of inflammatory factors are required. Insufficient MSC activation can lead to an increase in the inflammation [72]. Recently, it has been shown that IL-10 alone is insufficient to enhance MSC immunomodulation, rather enhances the priming influence of TNF-α, indicating that MSC activation by IL-10 is dependent on TNF-α [64]. MSCs further decrease TNF-α secretion via PGE2 but not IL-6, supporting the concept that MSC immunomodulatory potential is highly correlated to the release of PGE2 [64].

Among the other MSC-derived molecules shown to exert an immunoregulatory functions are transforming growth factor beta (TGF-β), hepatocyte growth factor (HGF), and indoleamine 2,3-dioxygenase (IDO) [73] (Fig. 2). TGF-β secreted by MSCs could shift lipopolysaccharide-activated macrophage polarization toward the M2-phenotype, decrease inflammatory reactions, and enhance the phagocytic activity through the Akt/FoxO1 pathway [74], while HGFs modulate IL-10 production in monocytes via the ERK1/2 pathway [75]. MSC IDO activity is involved in the differentiation of monocytes into IL-10-secreting M2 immunosuppressive macrophages (CD14^+^/CD206^+^) [71]. These processes decrease immune cell maturation and activation, in addition to enhancing the differentiation of T cells into regulatory T cells (Tregs) [52].

The immunoregulatory effects of MSCs is highlighted by the ability of BM-MSCs to suppress T cell proliferation [76, 77] and suppress the conversion of monocytes and CD34^+^ hematopoietic progenitor cells into dendritic cells (DCs) in vitro [78–81]. Mature DCs cultured with MSCs have reduced production of IL-12 and MHC class II molecules, CD11c, CD83, though hindering the DC antigen-presenting function [78–81].

### Anti-inflammatory and antiproliferative effects of MSCs

MSCs reduce the pro-inflammatory effect of DCs by suppressing their secretion of TNF [82]. Also, plasmacytoid DCs (pDCs), a set of specific cells for the secretion of high levels of type I IFN, increase the production of IL-10 following the incubation with MSCs [82]. MSCs can further inhibit the cytotoxic activity of resting NK cells by reducing the production of natural cytotoxicity receptor 3 (NKp30) and natural-killer group 2, member D (NKG2D), involved in the activation of NK cells and target cell killing [83]. Therefore, MSCs inhibit NK cell proliferation and IFN production [84, 85]. Also, neutrophils are important cells of innate immunity, undergoing a process known as the respiratory burst when binding to an antigen. MSCs have been reported to eliminate the respiratory burst and to prevent the neutrophil cell death by an IL-6-dependent mechanism [86]. Also, MSCs play a key role in the adaptive immune system, where it inhibits the proliferation of T cells activated with antigens [76]. This leads to a reduction in the IFN production and an increase in IL-4 production by T helper 2 (T2) cells, indicating a change in T cells from a pro-inflammatory (IFN-producing) to an anti-inflammatory (IL-4-producing) state [82].

Furthermore, MSCs have been shown to downregulate CD8^+^ cytotoxic T lymphocytes (CTL)-mediated cytotoxicity [87] and further inhibit B cell expansion in vitro. Also, MSCs can suppress B cell differentiation and the constitutive secretion of chemokine receptors, affected by the MSC-mediated suppression of T cell functions [88]. Furthermore, MSC-derived IDO has been shown to be required in the inhibition of the expansion of IFN-secreting Th1 cells and, together with PGE2, to stop NK cell activity [89].

### Anti-apoptotic and protective functions of MSCs

Several pro-inflammatory molecules modulate the immunosuppressive, trafficking, and paracrine potential of MSCs. Enhanced paracrine potential of MSCs induced by TNF-α, IL-1b, and nitric oxide (NO), ultimately increases MSC secretions of regenerative, immunomodulatory, and trafficking molecules, including the key factor, insulin-like growth factor 1 (IGF-1) [90]. Heme oxygenase-1 (HO-1) is upregulated by TNF-α, IL-1α, or NO in endothelial cells or alveolar cells, where MSCs overexpressing HO-1 showed an increase in the anti-inflammatory, anti-apoptotic, and vascular remodeling properties [91]. Upregulation of HO-1 increases production of trophic molecules, such as FGF2, and IGF-1, and VEGF [90]. Fibroblast growth factor-10 (FGF-10), keratinocyte growth factor-2 (KGF-2), has been found to regulate epithelial-mesenchymal interactions that are crucial for the development of lung [92]. FGF-10 exerts a role in lung resident-MSC propagation, mobilization, and the protective effects against acute lung injury [93]. MSCs can affect on the endothelial differentiation of endothelial progenitor cells in vitro, mainly dependent on VEGF [94]. Human leukocyte antigen-G5 (HLA-G5) is another soluble factor secreted by MSCs and its secretion is IL-10-dependent. HLA-G5 is required to suppress the function of T lymphocytes and NK cells and to activate regulatory T cells [95]. Galectin-1 and 3 (Gal-1 and Gal-3) as well as Semaphorin-3A (Sema-3A) are other secreted MSC immune regulators, known for their inhibitory activities. Gal-1 and Sema-3A are two soluble factors that can suppress T cell proliferation via neuropilin-1 (NP-1) binding [96, 97]. MSC-derived Gal-1 significantly regulates the release of TNFα, IFNγ, IL-2, and IL-10 [97].

Finally, interleukin-1 receptor antagonist (IL-1Ra) and programmed death-1 (PDL1) are among the other secreted regulators of MSCs. IL-1Ra is among the anti-inflammatory cytokine produced by MSCs, which can inhibit Th17 polarization. IL-1Ra expression tends to increase in MSCs exposed to IL-1β, TNF-α, and IFN-γ. Th17 cells induce the upregulation of PDL1, playing a major role in activating the MSC immunosuppressive effect [98]. PDL1 further support the cell-cell contact through MSC-mediated inhibition on Th17 cells [98]. MSC enhanced PDL1 ligand secretion suppress the activation of CD4^+^ T cells and downregulate IL-2 secretion [99].

### MSC-derived exosomes

Extracellular vesicle (EV) is a term including both exosomes and microvesicles (MVs). The exosome diameter is less than 200 nm, while MV diameter can reach up to 1000 nm. The secretomes of MSCs and their vesicles offer a powerful tool for cell-free therapy due to their paracrine and/or endocrine effects [100]. This strategy bypasses most of the safety concerns related to cell-based therapy, such as contamination with oncogenic cells and continuous cell proliferation [101]. The key features of MSC-derived EVs are (1) non-proliferative, which reduce the risk of tumor formation; (2) negative for HLA-I and HLA-II, which can be induced, and therefore, they can be used from other individuals without any risk of immune response; (3) small in size allowing them to pass from the small blood capillaries; and (4) stored without using DMSO, which may change their characteristics [102]. EVs bind to a receptor on the cell membrane of the targeted cells, where they merge with the membrane to secrete the EV contents inside the cell or enter into the cytoplasm in the form of endocytic vesicles [103].

EVs have proposed as an effective vehicle for delivering miRNAs, which control above 60% of the mRNAs; therefore, transferring them in EVs is of clinical significance [102]. MSC engineering is one way miRNA could be loaded into EVs and still exert its therapeutic effects [104–113]. There is a lack of a consensus on miRNA signature among MSC-EVs from various sources [114]. However, the targeted pathways include Wnt signaling, antifibrotic, mitochondrial fission, cell proliferation, cell survival, and apoptosis [115]. Reports showed that the MSC-EV-mediated delivery of miRNAs in animal models have defined several key target proteins like TGF-β receptor 1, Dynamin-related protein 1(DRP1), Methyl-CpG-binding protein 2 (Mecp2), PTEN, semaphorin 3A (sema3A), stat3, Cyclin G1, IGF1R, and P4HA1, NLRP3, and Bcl-2 [9, 104–113] (Table 1).

**Table 1:** Studies demonstrating the MSC-EV-mediated transfer of miRNAs in animal models

miRNA transferred	Target proteins	Function	Reference
miR-let7c	TGF-β receptor 1	Anti-fibrotic	[104]
miR-30	Dynamin-related protein 1 (DRP1)	Regulate mitochondrial fission	[105]
miR-22	Methyl-CpG-binding protein 2 (Mecp2)	Anti-fibrotic	[106]
miR-19a	PTEN	Cell survival signaling pathway	[107]
miR-223	Semaphorin 3A (Sema3A) and Stat3	Anti-apoptotic and antiinflammatory	[108]
miR-122	Cyclin G1, IGF1R, and P4HA1	Anti-proliferative and antifibrotic	[109]
miR-223	NLRP3	Anti-inflammatory: decrease pytoptosis and IL-1β	[110]
miR-181	Bcl-2 and Stat3	Anti-fibrotic and activated autophagy	[111]
miR-133	RhoA and connective tissue growth factor	Enhanced plasticity	[112]
miR-17-92	PTEN	Cell survival signaling pathway	[113]

*IGF1R* insulin-like growth factor receptor 1, *P4HA1* prolyl 4-hydroxylase alpha 1, *NLRP3* NLR pyrin domain-containing 3, *RhoA* homolog gene family member A, *BCL-2* B cell lymphoma 2 family

Among the targeted proteins, Sema3A has been found to induce sepsis-triggered cytokine storm through an interaction with Plexin-A4 and Toll-like receptors (TLRs) [116]. Stat3 is another targeted protein, a key upstream stimulator of inflammatory pathways during sepsis [117]. Finally, EVs act as biological regulators that can promote changes in their targets through targeted pathways. The cargo of the EVs is enriched with miRNAs and other transcripts that act as regulators of the immune system [118, 119]. Therefore, EVs are attractive tools for clinical applications as immunosuppressants, vaccines, or activators of differentiation and repair processes [120].

## MSCs and their exosomes as potential therapies for COVID-19

MSCs have been well described in ALI and ARDS. It exerts its function via targeting both infectious, inflammatory, and endothelial factors. MSCs can release KGF2, PGE2, GM-CSF, IL-6, and IL-13 to facilitate phagocytosis (Figs. 2 and 3). In addition, multiple clinical studies [121–125] investigated the effect and mechanism of MSCs and MSC-EVs on lung injuries caused by different reasons (Table 2). MSCs and their secreted secretome exert an immunomodulatory, anti-inflammatory, anti-apoptotic, and anti-fibrotic functions in ALI and ARDS. PGE2 changes the macrophage polarization from M1 to M2 [144], IL10 decreases the recruitment of the neutrophils into the lung [145], and IDO enhances pulmonary antimicrobial activity [146]. Furthermore, the propagation, differentiation, and chemotactic features of B cells are hindered by MSCs as well [147] (Fig. 2). MSCs can further enhance restoration of capillary barrier, restore alveolar ATP [141], where the secreted growth factors KGF, VEGF, and HGF, can exert a protective effect on the alveolar cells [148]. In ALI models, the KGF mRNA has been involved in the immunomodulation noticed with MSC-EV treatment [126, 129]. MSC anti-bacterial effect is further demonstrated in inhibition of bacterial growth [57]. Several preclinical studies examined the therapeutic effects of MSCs and MSC-derived EVs in animal models of ALI, ARDS, and other lung inflammatory conditions [126–143, 149–151] (Table 2). These studies showed a significant decrease in the inflammatory reactions, improved edema clearance, and restored epithelial damage (Table 2). A preclinical study reported that the intratracheal administration of MSCs increases the accessibility of MSCs to both the alveolar epithelium and the pulmonary endothelium [152], where MSCs demonstrate reduction in endotoxin-induced injury to explanted human lungs [153].

**Table 2:** Biological effect and molecular mechanisms of MSCs and MSC-EVs in preclinical and clinical studies looking into lung injury

Disease	Study and/or cell type	Postulated Mechanism of MSC action	Route of MSC and/or MSC-MV administration	EV isolation	Reference
**Clinical studies**					
ARDS	- RCT pilot study - Allogeneic AT-MSCs	- Decrease in surfactant protein D (SP-D) - Decrease in Il-6, Il-8 (not statistically significant)	- IV dose of 1 × 10^6^ cells/kg	N.A.	[121]
Bronchopulmonary dysplasia (BPD)	- Phase I dose-escalation trial - UC-MSCs	- Reduction of IL-6, IL-8, MMP-9, TNF-α, and TGF-β1 in tracheal aspirates at day 7	- Intratracheal administration - In nine preterm infants. - The first three patients were given a low dose (1 × 10^7^ cells/kg) of cells - The next six patients were given a high dose (2 × 10^7^ cells/kg)	N.A.	[122]
COPD	- RCT pilot study - Allogeneic MSCs (Prochymal; Osiris Therapeutics Inc.)	- Decrease in levels of circulating CRP (significant) - Levels of circulating TNF-α, IFN-γ, IL-2, IL-4, IL-5, and IL-10 were at or below limits of assay detection (preventing meaningful analysis) - Levels of circulating TGF-β and CRP did not differ significantly between baseline to years 1 or 2 in either treatment group	- 62 patients were randomized to double-blinded IV infusions - Patients received four monthly infusions (100 × 10^6^ cells/infusion) and were subsequently followed for 2 years after the first infusion	N.A.	[123]
ARDS	- The START trial was a multi-center, open-label, dose-escalation phase 1 clinical trial - BM-MSCs	- Decrease in IL-6, RAGE, and Ang-2 levels (dose-independent)	- Three patients were treated with low dose MSCs (1million cells/kg), IV - Three patients received intermediate dose MSCs (5 million cells/kg), IV - Three patients received high dose MSCs (10 million cells/kg, IV)	N.A.	[124]
ARDS	- Non-randomized, pilot study (2 patients) - BM-MSCs	- Decrease in ccK18 and K18 - Decline in pro-inflammatory miRNAs in circulating EVs (miR-409-3P, 886-5P, 324-3P, 222, 125A-5P, 339-3P, 155) - Increased levels of circulating CD4+CD25^high^CD127^low^ T_Regs_ were observed in both patients’ peripheral blood	- 2 × 10^6^ cells/kg IV	N.A.	[125]
**Preclinical studies**					
ALI (endotoxin induced/*E. coli*)	Human BM-MSC	- Reduction in neutrophils and MIP-2 levels in the BAL - KGF-expressing MV transfer to injured alveolus - Reduced EVLW, improved lung endothelial barrier permeability and restored alveolar fluid clearance - -Restoration of the total cellular level and the apical membrane expression of αENaC	- 30 μl of MVs released by 1.5–3 × 10^6^ serum starved MSCs - IT and IV routes - Ex vivo human lung and Human AT2 Cells. - IT dose: 750,000 MSCs	UCF (3000 rpm/Beckman Coulter Optima L-100XP)	[126]
ARDS (*E. coli* endotoxin)	Human BM-MSCs	- Increased M2 macrophage marker expression (CD206) - increased phagocytic capacity - EV-mediated mitochondrial transfer	- Ex vivo (murine) - EVs released by 15 × 10^6^ MSCs over 48 h	UCF (10,000–100,000 xg)	[127]
Caecal ligation and puncture sepsis model (lung injury)	- Human UC-MSCs (IL-1β pretreatment)	- Induced M2 polarization - Exosomal miR-146a transfer to macrophages	- IV - 30 μg exosomes - 1 × 10^6^ MSCs	UCF (Beckman Optima L-80 XP)	[128]
*E. coli* pneumonia-induced ALI	Human BM-MSCs	- KGF-expressing EV transfer/CD44 receptor dependent - Increased monocyte phagocytosis (antimicrobial) - Reduced the total bacterial load, inflammation, and lung protein permeability in the injured alveolus in mice - Decreased TNF- - Restoration of intracellular ATP levels in injured human AT2 (primary human AT2 culture) - TLR3 prestimulation increased mRNA expression for COX2 and IL-10	- 10 μl per 1 × 10^6^ MSCs - 30 or 60 μl MV, instilled IT - 90 μl MV, injected IV	UCF	[129]
Silicosis-induced lung injury/silica-exposed mice	- Human BM-MSCs - Mouse MSCs	- EVs outsource mitophagy, improve mitochondria bioenergetics via ARMMs - Represses TLR signaling in macrophages - Repress the production of inflammatory mediators via TLRs and NF-kB pathway (miR-451) - Prevent the recruitment of Ly6C^hi^ monocytes and reduces IL-10 and TGF-β secretion (pro-fibrotic) by these cells in the lung of silica-exposed mice	- 40 μg protein (3 × 10^11^ EVs), IV	UCF	[130]
Emphysema/elastase-induced COPD model	Human AD-MSCs	- EV transfer to alveolar epithelium-FGF2 signaling	- IT - 1 mg nanovesicle from 7 × 10^7^ ASCs (30 × 10^6^ nanovesicle generated)	UCF (100,000×*g* force). Nanovesicle 100-nm	[131]
ALI (HPH)	- Mouse BM-MSCs - Human UC-MSCs	- EV transfer to endothelial cells suppress STAT3 signaling - Upregulation of the miR-17 superfamily of microRNA clusters - increased lung levels of miR-204 - Suppress pulmonary influx of macrophages	- IV - 0.1–10 μg MSC-derived exosomes	UCF (100 kDa cut-off/Millipore)	[132]
PAH	- Murine MSC(mMSC) - Human BM-MSCs	- Prevent and reverse pulmonary remodeling via EV miRNA transfer - Increased levels of anti-inflammatory, anti-proliferative miRs including miRs-34a, -122, -124, and -127.	- 25 μg of MVs, IV	UCF (100,000×*g*)	[133]
BPD (hyperoxia)	- Human UC-MSC - Human BM-MSCs	- Reduced mRNA levels of pro-inflammatory M1 macrophage markers (Tnfa, Il6, and Ccl5). - Enhanced M2 macrophage marker (Arg1) - Suppressed the hyperoxic induction of Cd206 - Significantly suppressed Retnla	- 0.9–3 μg protein, IV	UCF (OptiPrep/EVs 30–150 nm)	[134]
BPD (hyperoxia)	Human UC-MSCs	- TSG-6-expressing EV transfer - Decrease in IL-6, TNF-α, and IL-1β	- 2.4–2.8 μg EVs (obtained from 0.5–1 × 10^6^ MSC), IP	UCF	[135]
Bleomycin (BLM)-induced lung inflammation and fibrosis	- Mouse BM-MSCs - Human BM-MSCs	- Block upregulation of IL-1 gene expression - IL1RN expressed by MSCs blocks release of TNF-α from activated macrophages - IL1RN is the principal IL-1 antagonist secreted by murine MSCs	- 5 × 10^5^ MSCs, IV	N.A.	[136]
ALI (endotoxin induced)	Mouse-BM-MSCs	- Decreased total WBCs, neutrophils, MIP-2, EVLW, and TNFα - Increase expression of KGF mRNA in the injured alveolus - Increase IL-10	- IT MSCs administration - 20,000 cells/100 μl for co-culture in vitro and transwell	-Transwell	[137]
ALI (primary human AT2)	Allogeneic human BM-MSCs	- Suppression of NFκB activity and further cytoskeletal re-organization of both actin and claudin 18 - Increase secretion of paracrine soluble factors angiopoietin-1 and Tie2 phosphorylation - Restoration of type II cell epithelial permeability to protein (Alveolar barrier integrity)	- Alveolar epithelial type II	Transwell plate	[138]
Pneumonia (*E. coli*)	Mouse BM-MSCs	- Decrease level of MIP-2 and TNFα, neutrophil degranulation in the alveolar space - Upregulate the concentration of lipocalin 2 expression (antimicrobial factor) in the alveolar space	- IT - 750,000 MSCs	N.A.	[139]
Pneumonia (*E. coli*)	Human MSCs	- MSC preferentially migrated to endotoxin-injured lung tissue - Increase KGF secretion - Human monocytes expressed the keratinocyte growth factor receptor - Reduced apoptosis of human monocytes through AKT phosphorylation - Increased the antimicrobial activity of the alveolar fluid (alveolar macrophage phagocytosis). - Decrease in TNF-α - Increase in IL-10	- 5–10 × 10^6^ human MSC, was instilled IB or IV (human ex vivo and in vitro monocyte studies)	N.A.	[140]
ALI (LPS-induced)	Mouse-BM-MSCs, human BM-MSCs	- Connexin 43-dependent mechanisms and transfer of viable mitochondria	- 2 × 10^5^ BM-MSCs IT	N.A.	[141]
Acute lung injury	Rat-BM-MSCs	- Attenuated alveolar TNF α - Increase IL 10	- 2 × 10^6^ cells of MSCs, IV	N.A.	[142]
Acute lung injury	Clinical-grade human allogeneic-BM-MSCs	- Reduction in the airspace levels of RAGE, a marker of AT1 injury/activation - Increase secretion of KGF	- Ex vivo lung perfusion model (5 × 10^6^ cells hMSCs, IB)	N.A.	[143]

*RCT* randomized, placebo-controlled; *MSC*, mesenchymal stem cell; *ILD* interstitial lung disease; *ARDS* acute respiratory distress syndrome; *START* the stem cells for ARDS treatment; *ALI* acute lung injury; *IPF* idiopathic pulmonary fibrosis; *COPD* chronic obstructive pulmonary disease; *HPH* hypoxia-induced pulmonary hypertension; *PAH* pulmonary artery hypertension; *BPD* bronchopulmonary dysplasia; *BM* bone marrow; *UC* umbilical cord; *AD* adipose tissue;, *MMP-9* matrix metalloproteinase-9; *Ang-2* angiopoeitin-2; *RAGE* receptor for advanced glycation end products; *ccK18* caspase-cleaved cytokeratin-18; *K18* cytokeratin-18; *KGF* keratinocyte growth factor; *TGF-β1* transforming growth factor beta 1; *TSG-6* tumor necrosis factor alpha-stimulated gene-6; *UCF* ultracentrifugation; *IL1RN* interleukin 1 receptor antagonist; *AT1* Alveolar epithelial type I; *AT2* Alveolar epithelial type II; *AT-MSCs* adipose-derived MSCs; *hWJMSC* human umbilical cord Wharton’s jelly MSC; *IB* intrabronchially; *IT* intratracheal; *IV* intravenous; *IP* intraperitoneal; *BAL* bronchoalveolar lavage; *MIP-2* Macrophage Inflammatory Protein 2; *EVLW* extravascular lung water; *STAT3* signal transducer and activator of transcription 3; *IL-1β* interleukin-1β; *TLR3* toll-like receptor-3; *COX2* prostaglandin-endoperoxide synthase 2; *ARMMs* arrestin domain-containing protein 1-mediated MVs; *ASCs* adipose-derived stem cells; *IL1RN* interleukin 1 receptor antagonist; *WBCs* white blood cells; *RAGE* receptor for advanced glycation end products

MSC-derived exosomes are a multitargeted biologic agent, which can reduce the cytokine storm and reverse the inhibition of host anti-viral defenses associated with COVID-19 [154]. The functions of the MSC-derived exosomes have been studied in in vitro and in vivo models. EVs collected from the conditioned medium of BM-MSCs have been used to treat ARDS in a mouse model. The results showed that EVs have a similar effect as MSCs in reducing the inflammation and edema in the lung [126]. The effect of MSCs on macrophage modulation in ARDS mouse models has been mainly found due to the effect of EVs [127]. Treating mouse alveolar macrophages with MSC-EVs has a protective effect in the mouse model of lipopolysaccharide (LPS)-induced lung injury [127]. It has been found that the transferred active mitochondria through EVs leads to a change in the polarization of the macrophage from M1 (pro-inflammatory) to M2 (anti-inflammatory) phenotype as a result of an increase in the oxidative phosphorylation [127]. Also, it has been reported that functional mitochondria transferred through MSC-EVs enhances mitochondrial function in primary human alveolar cells and enhances their ability to repair lung injuries [155]. In addition, the effect of MSC-EVs has been examined in pneumonia mouse model induced by *E. coli* [129]. The data showed that EVs could decrease lung inflammation by reducing neutrophil and macrophage recruitment as well as MIP-2 level [129]. It has been found that the EVs reduce lung edema and endothelial permeability and the expression of CD44 on the target cells are required for the binding and uptake of EVs into cells [129]. BM-MSC-EVs have been used in another ARDS mouse model induced by LPS from *Pseudomonas aeruginosa* [151]. Tang et al. reported that the EV-mediated transfer of angiopoietin-1 (Ang1) mRNA is important for inflammation reduction, endothelial cell protection, and barrier repair through decreasing neutrophil influx and MIP-2 level [151]. Furthermore, EVs exert an immunomodulatory function in the macrophage by inhibiting the secretion of TNF-α and enhancing the secretion of IL-10 [151]. In a pig model, the influence of MSC-EVs on influenza virus-induced ARDS has been investigated [155]. Administration of EVs has been found to decrease the influenza virus replication, pro-inflammatory cytokines, and alveolar cell death in pigs through the transfer of RNA [155]. UC-MSC-EVs have been also used in a rodent model. The study found that the UC-EVs are effective in reducing ALI and the EVs primed with INF-γ are more efficient than normal EVs in improving ALI [156]. This indicates that EVs isolated from different source could be used for lung injury. Interestingly, the primed EVs have been found to be larger in size than normal EVs; however, the mechanism of this size increase remains unclear [156]. Previous studies used in vitro human injured lungs to investigate the effect of MSC-EVs. MSC-EVs restore fluid clearance and reduce edema in human injured lungs in vitro [143, 157]. Another study examined the effect of EVs on human lungs with pneumonia induced by *E. coli* found that EVs reduce the permeability of lung protein and enhance alveolar fluid clearance [158]. Barrier properties of the of human lung endothelial cells injured with TNF-α, IFN-γ, and IL-1β are restored with EVs. This improvement is due to an increase in the levels of Ang-1 in the injured endothelium, treated with EVs [159]. Although there are promising results obtained from using MSC-EVs in lung injury, more mechanistic studies are needed to improve our understanding on the molecular mechanisms involved in EV effect.

MSCs could act upon two ways in the novel COVID19 treatment, namely via its immunomodulatory effects and differentiation ability. MSCs display numerous advantages of relevance to ALI and ARDS. Although progress in the management of ALI/ARDS depends on improvements in supportive measures, ultimately decreasing the mortality rates [160], the failure of pharmacologic treatments indicate the need to consider new strategies for ALI/ARDS. MSC possible therapeutic potential is attributed to their accessible derivation from several adult tissues, their low immunogenicity, indicating that they could be given allogeneically [161], and their relative ease of isolation and expansion ability in culture. In case of COVID-19 patients, autologous and allogenic MSC transplantation could be applied, because MSCs do not express ACE2 and TMPRSS2; therefore, patient’s own MSCs cannot be infected by SARS-CoV2 [162]. However, the negative effects caused by the SARS-CoV2 infection on the blood cells and different organs may influence the ability to isolate autologous MSCs of high quality and sufficient number to treat the same patient. Taken together with the low immunogenicity of MSCs and the complications associated with the SARS-CoV2 infection, using allogenic MSCs is the method of choice for COVID-19 patients.

Resolution of ALI/ARDS in COVID19 is hindered by the disruption of the epithelial barrier that suppresses alveolar fluid clearance and depletes surfactant [163]. MSC capacity to aid in restoring epithelial and endothelial function by differentiating MSCs into these cell types or by secreting paracrine and trophic factors to increase restoration of the lung tissue offers a promise for treatment of ALI/ARDS in COVID19. MSCs have been widely studies in other inflammatory conditions, where they demonstrated a reduction in injury and/or enhanced restoration of function in the kidney [164, 165], liver [166], and heart [167]. MSC immunomodulatory properties exhibit a promise for treating ALI/ARDS in COVID19 via their ability to ‘reprogramme’ the immune response to decrease the destructive inflammatory components, while maintaining the host response to infections, in addition to enhancing the repair and resolution of lung injury by acting an effector for tissue regeneration.

## MSC clinical trials for COVID-19 patients

With nearly 67 registered clinical trials in ClinicalTrials.gov looking into the use of MSC/MSC-derived EVs in ARS-CoV-2 associated disease, only few have been published (Tables 3 and 4). To date, four articles have reported results of COVID-19 pneumonia treatment with MSCs. Liang et al. have demonstrated the safety and efficacy of human UC-MSCs in modulating the immune response and recovered the disrupted tissue of a 65-year-old female severally sick COVID-19 patient [168]. The patient received IV infusion of MSCs three doses (5 × 10^7^ cells/dose, every 3 days), in which following the second dose, clinical improvement has been observed. Furthermore, the number of the neutrophils and inflammatory cells in the patient reduced to a normal level, while the number of lymphocytes elevated to their normal levels [168]. Also, a recent study reported that IV injection of clinical-grade MSCs (1 × 10^6^/kg) into seven patients with ARS-CoV-2 leads to an improvement in the functional outcomes and a recovery enhancement [162]. Among the beneficial outcomes of MSC treatment are an observed increase in the number of peripheral lymphocytes, a decline in the C-reactive protein (CRP), a decrease of overactivated cytokine-producing immune cells (CXCR3^+^CD8^+^ T cells, CXCR3^+^CD4^+^ T cells, and CXCR3^+^ NK cells) and TNF-α, and increase in IL-10, mainly attributed to the anti-inflammatory and immunomodulatory functions of MSCs [162]. Furthermore, an increase in CD14^+^CD11c^+^CD11b^mid^ regulatory DC population has been observed [162]. Leng et al. demonstrated that MSCs modulate the lung microenvironment by protecting or rejuvenating alveolar epithelial cells, reducing fibrosis, and enhancing pulmonary function [162]. RNA-seq analysis for the transplanted MSCs in COVID-19 patients showed that the transplanted MSCs do not express ACE2 or TMPRSS2, indicating that MSCs cannot be infected with COVID-19; however, they express high levels of anti-inflammatory and paracrine factors, such as HGF, FGF, EGF, TGF-β, GAL, LIF, NOA1, VEGF, NGF, and BDNF. Also, the transplanted MSCs express high levels of AT2-specific surfactant proteins, SPA and SPC, suggesting that the MSCs may differentiate into AT2 cells [162].

**Table 3:** Ongoing clinical trials using MSCs and MSC-derived exosomes to treat COVID-19 patients

Clinical trial identifier	Study design	Phase	Intervention/treatment	Dose and route of administration	Estimated enrollment	Control group	Country	Recruitment status
NCT04348461^#^	RCT, parallel assignment, multicenter, quadruple* masking	2	Allogeneic AT-MSCs	Two doses of 1.5 × 10^6^/kg, IV	100 (50 each group)	Standard of care	Spain	Not yet recruiting
NCT04467047	Interventional, open label	1	Allogenic BM-MSCs	1 × 10^6^ MSCs/kg, IV	10	None	Brazil	Not yet recruiting
NCT04473170	RCT, open-label	1/2	Autologous NHPBSCs	Dose: non specified, jet nebulization	146	Standard care	UAE	Completed
NCT04349540	Prospective non-interventional study	NA	Allogenic hematopoietic stem cells	Not defined	40	None	UK	Active, not recruiting
ChiCTR2000029990^#^	Pilot trial, interventional study	2	MSCs (undefined source)	1 × 10^6^ per kg of weight, IV	7 patients for MSC transplant and 3 for placebo	Vehicle placebo	China	Recruiting
NCT04466098	Interventional, randomized, placebo-controlled, parallel assignment, triple masking (participant, care provider, investigator)	2	MSCs (undefined source)	300 × 10^6^ MSCs, three fixed doses of MSCs, approximately 48 h apart, IV	30 randomized (2:1 ratio) placebo-controlled trial.	Vehicle placebo (Dextran 40 + 5% human serum albumin)	USA	Recruiting
NCT04445220	Interventional, randomized, placebo-controlled, parallel assignment, quadruple masking (participant, care provider, investigator)	1/2	Allogeneic human MSCs (undefined source)	Low dose cohort: SBI-101 device containing 250 million MSCs; high dose cohort: SBI-101 device containing 750 million MSCs, extracorporeal	24	Sham device containing no MSCs	USA	Not yet recruiting
NCT04447833	Single group assignment, open label	1	Allogeneic BM-MSCs	First three patients receive a single dose of 1 × 10^6^ MSCs/kg dose, next six patients receive a single dose of 2 × 10^6^ MSCs/kg, IV	9	N.A.	Sweden	Recruiting
NCT04457609	RCT, parallel assignment, triple*	1	Allogenic UC-MSCs	Intravenous infusion of 1 × 10^6^ unit of UC-MSCs/kg BW in 100 cc of 0.9% NaCl for 1 h, in addition to standardized treatment (oseltamivir and azithromycin)	40	Placebo (oseltamivir and azithromycin)	Indonesia	Recruiting
NCT04397471	Observational, prospective	N.A	Allogenic BM-MSCs	Not defined	10	N.A.	UK	Not yet recruiting
NCT04461925	Non-randomized, parallel, open label	1/2	Allogenic placenta-derived MSCs	1 million cells/kg body weight/time, once every 3 days for a total of 3 times: day “1”, day “4”, day “7”, IV + ceftriaxone, azithromycin, anticoagulants, hormones, oxygen therapy, mechanical ventilation and other supportive therapies	30	Ceftriaxone, azithromycin, anticoagulants, hormones, oxygen therapy, mechanical ventilation and other supportive therapies	Ukraine	Recruiting
NCT04428801	RCT, double* masking	2	Autologous AT-MSCs (Celltex-AdMSCs)	200 million every 3 days (3 doses), IV	200	Placebo (not defined)	USA	Not yet recruiting
NCT04416139	Non-randomized, parallel assignment, open label	2	MSCs (undefined source/from bank)	1 million/kg in a single dose, IV	10	Standard management measures	Mexico	Recruiting
NCT04429763	RCT, parallel design, triple* masking	2	Allogenic UC-MSCs	1 × 10^6^ cells/kg single dose, IV	30	Placebo (not defined)	Colombia	Not yet recruiting
NCT04444271	Interventional, RCT, parallel design, open label	2	Autologous BM-MSCs	2 × 10^6^ cells/kg on days 1 and 7 (if needed), IV	20 (20 each group)	Placebo (100 ml normal saline IV)	Pakistan	Recruiting
NCT04456361	Interventional, single group assignment, open label	1	WJUC-MSCs	Single-dose of 1 × 10^8^ cells, IV	9	N.A.	Mexico	Active, not recruiting
NCT04366271	Randomized, interventional, parallel design, open label	2	Allogeneic UC-MSCs	Single IV infusion MSCs (dose unspecified) + standard of care	10^6^	N.A.	Spain	Recruiting
NCT04371393	RCT, parallel design, triple* masking	3	Allogenic BM-MSCs (Remestemcel-L) + standard of care	Two doses of 2 × 10^6^ MSC/kg (doses 4 days apart ± 1 day), IV+ standard of care	300 (150 each group)	Placebo (Plasma-Lyte + standard of care)	USA	Recruiting
NCT04313322	Interventional, prospective, single group, open-label.	1	Allogenic WJ-MSCs	Three doses of 1 × 10^6^/kg, 3 days apart, IV	5	N.A.	Jordan	Recruiting
NCT04452097	Interventional, prospective, single group, open-label.	1	UC-MSC	0.5 million cells/kg, IV, plus standard treatment	9	N.A	NA	Not yet recruiting
NCT04315987	RCT, quadruple* masking	2	NestCell® + standard of care	Four doses of 2 × 10^6^/kg, at days 1, 3, 5, and 7, IV	90 (45 each group)	Placebo (undefined)	Brazil	Not yet recruiting
NCT04252118 (preliminary for NCT04288102)	Interventional, prospective, non-randomized, parallel assignment, open-label	1	Allogenic UC-MSCs + conventional treatment	Three doses of 3.0 × 10^7^ at days 0, 3, and 6	20 (10 patients in each arm)	Conventional treatment	China	Recruiting
NCT04288102	RCT, quadruple* masking	2	Allogenic UC-MSCs + conventional treatment	Three doses of 4 × 10^7^, at days 0, 3, 6, IV + standard of care	90 (60 patients assigned to treatment and 30 to control group)	Placebo (saline containing 1% human serum albumin)	China	Completed
NCT04302519	Interventional, prospective, non-randomized, single group, open-label	1	Dental pulp MSCs + conventional treatment	Three doses of 1.0 × 10^6^ cells/kg, at days 1, 3, and 7, IV	24	N.A.	Shanghai	Not yet recruiting
NCT04273646	RCT, parallel assignment, open label	N.A.	Allogenic UC-MSCs + conventional treatment	Four doses of 5.0 × 10^6^ cells/kg at, days 1, 3, 5, and 7, IV+ conventional treatment	48 (24 patients in each arm)	Placebo + conventional treatment	China	Not yet recruiting
NCT04299152	Prospective, two-arm, partially masked/single masking (care provider).	2	Preconditioned CB-MSC (patient mononuclear cells)	N.A.	20	Conventional treatment	China	Not yet recruiting
NCT04269525	Interventional, prospective non-randomized, single group assignment, open-label.	2	Allogenic UC-MSCs	Four doses of 3.3 × 10^7^cells at, days 1, 3, 5, and 7, IV	10	N.A.	China	Recruiting
NCT04333368	Interventional, RCT, parallel assignment, triple* masking	1/2	Allogenic WJUC-MSCs + standard of care	Three doses of 1.0 × 10^6^ cells/kg at days 1, 3, and 5, IV	40 (20 treated, 20 placebos)	Placebo (NaCl 0.9%) + standard of care	France	Recruiting
NCT04276987	Interventional, prospective, single group assignment, open-label	1	Allogeneic AT-MSCs-Exo + conventional treatment	Five doses of 2.0 × 10^8^ nanovesicles, days 1, 2, 3, 4, and 5, aerosol inhalation route	30	N.A.	China	Completed
NCT04336254	Interventional, RCT, triple* masking	1/2	Allogeneic human dental pulp MSCs	Three doses of 3.0 × 10^7^ cells/dose at, days 1, 4 and 7, IV	20	Placebo (3 ml 0.9% saline)	China.	Recruiting
NCT04348435	RCT, double-blinded	2	Allogeneic AT-MSCs (HB-adMSCs)	Five doses of either 2 × 10^8^, 1 × 10^8^ or 5 × 10^7^ cells/single doses at weeks 0, 2, 6, 10, and 14, IV	100	Placebo (saline)	USA	Enrolling by invitation
NCT04352803	Non-randomized, sequential assignment, open-label	1	Autologous AT-MSCs	5 × 10^5^/kg, IV	20	N.A.	N.A.	Not yet Recruiting
NCT04366323	RCT, parallel assignment open-label	1/2	Allogeneic AT-MSCs	Two doses of 8 × 10^7^ cell/dose, IV	26	No intervention	Spain	Recruiting
NCT04349631	Interventional, single group assignment, open-label	2	Autologous AT-MSCs	Five doses of cells (unspecified dose), IV	56	No	USA	Enrolling by invitation
NCT04346368	RCT, single masking (participant).	1/2	BM-MSCs + conventional treatment	Single dose 1 × 10^6^ MSCs/kg, IV	20	Placebo + conventional treatment	China	Not yet recruiting
NCT04382547	Non-randomized, parallel assignment, open label	1/2	Allogeneic Om-MSCs + conventional treatment	Doses N.A., IV	40	Conventional treatment	Belarus	Enrolling by invitation
NCT04366063	RCT, parallel assignment, open-label	2/3	MSCs (undefined source) and EV-MSCs + conventional treatment	Intervention group 1: two doses 1 × 10^8^ at day 0, 2, IV Intervention group 2: two doses 1 × 10^8^ at day 0, 2 + EVs at days 4, 6, IV	60 (20 into two intervention groups, 20 control)	Conventional treatment	Iran	Recruiting
NCT04437823	Randomized, parallel assignment	2	UC-MSCs	5 × 10^5^ UCMSCs per kg, IV on days 1, 3 and 5 besides the standard care (SOC)	20	SOC	Pakistan	Recruiting
NCT04339660	RCT, triple* masking	1/2	Allogeneic UC-MSCs	One-two doses of 1 × 10^6^/kg (1 week apart), IV	30 (15 each group)	Placebo (saline)	China	Recruiting
NCT04392778	Interventional, RCT quadruple* masking	1/2	Allogeneic UC-MSCs	Three doses of 3 × 10^6^ cells/kg on days 0, 3, and 6, IV	30 (10 each group)	Placebo (saline)	Turkey	Recruiting
NCT04371601	RCT, Open-label	1	Allogeneic UC-MSCs + oseltamivir	Four single doses of 1 × 10^6^/kg, 4 days apart, IV + Oseltamivir	60	Oseltamivir	China	Active, not recruiting
NCT04355728	RCT, parallel assignment, double blinded,	1/2	Allogeneic UC-MSCs as add-on therapy + standard of care	Two doses of 100 × 10^6^ cells, IV	24 (12 each group)	Standard of care	USA	Recruiting
NCT04362189	RCT, quadruple* masking	2	Allogeneic AT-MSCs	Four doses of 1.0 × 10^8^ cells at days 0, 3, 7, and 10, IV	100 (50 each group)	Placebo (saline)	USA	Not yet recruiting
NCT04390152	RCT, quadruple* masking	1/2	Allogeneic WJ-MSCs + standard of care	Two doses of 50 × 10^6^, IV	40	Hydroxychloroquine, lopinavir/ritonavir or azithromycin and placebo (standard therapy)	Colombia	Not yet recruiting
NCT04377334	Randomized, parallel assignment, Open-label	2	Allogeneic BM-MSCs	N.A.	40 (20 each group).	No intervention.	Germany	Not yet recruiting
NCT04331613	Interventional, open-label	1/2	Human embryonic stem cell-derived M cells (CAStem)	Doses of 3, 5, or 10^6^ cells/kg, route not specified	9	N.A	China	Recruiting
NCT04390139	RCT, quadruple* masking	1/2	WJ-MSCs + standard of care	Two doses, 1 × 10^6^ cells/kg, IV	30 (15 to each group)	Placebo + standard of care	Spain	Recruiting
NCT04400032	Interventional, non-randomized, sequential assignment, open-label	1	BM-MSCs (undefined source)	Intervention group 1: three doses 25 × 10^6^ at day 0, 1, 3 IV Intervention group 2: three doses 50 × 10^6^ at day 0, 1, 3 IV Intervention group 3: three doses 90 × 10^6^ at day 0, 1, 3, IV	9	N.A.	Canada	Not yet recruiting
NCT04398303	RCT, double* masking	1/2	Allogeneic WJ-MSCs and WJ-MSC-CM	Intervention group 1: 1.0 × 10^6/^/kg cells in 100 ml CM+ Conventional treatment Intervention group 2: 100 ml CM+ conventional treatment	70	Conventional treatment |+placebo	USA	Not yet recruiting
NCT04365101	RCT, open-label	1/2	Natural killer (NK) cells derived from human placental CD34^+^ cells	CYNK-001 infusions on days 1, 4, and 7	86 (1:1 randomization ratio)	Best supportive care	USA	Recruiting
NCT04393415	Randomized, parallel, double masking	N.A.	Allogeneic UC-MSCs	UC-MSC (undefined dose) + platelet rich plasma (PRP)	100	PRP.	Egypt	Not yet recruiting
NCT04397796	RCT, quadruple* masking	1	Allogeneic BM-MSCs	Undefined dose and route	45	Placebo (plasmalyte and albumin)	USA	Not yet recruiting
NCT03042143	RCT, quadruple*masking	1/2	Allogeneic WJ-MSCs (CD362 enriched)	Single dose of 4 × 10^8^ cells, IV	75	Placebo (plasmalyte)	UK	Recruiting
NCT04345601	Single group, parallel assignment, open-label	1	Allogeneic BM-MSCs	Single dose of 1X10^8^ cells, IV	30	Standard of care	USA	Not yet recruiting
NCT04361942	RCT, Triple*masking	2	Allogeneic MSCs	Single dose of 1 × 10^6^ cells/kg, IV	24	Placebo (saline)	Spain	Recruiting
NCT04333368	RCT, Triple*masking	1/2	Allogeneic WJ-MSCs	Three doses of 1 × 10^6^ cells/kg at days 1, 3, and 5, IV	40 (20 each group)	Placebo (0.9% saline)	France	
NCT04389450	RCT, quadruple* masking	2	Allogeneic PLX-PAD	High and low doses groups (cell dose unspecified), 1 week apart, IM	140	Placebo	USA	Recruiting
NCT04367077	RCT, sequential assignment, quadruple* masking	2/3	BM-MSCs (MultiStem), source unspecified	Dose unspecified, IV	400	Placebo	USA	Recruiting

^#^Trial with published results, and withdrawn trials were excluded from the table

*ChiCTR* Chinese Clinical Trial Registry; *NCTnumber* ClinicalTrials.gov identifier; *RCT* Randomized control trial; Triple* masking participant, investigator, outcomes assessor; Double* masking participant, outcomes assessor; *MSCs* mesenchymal stem cells; *NHPBSC* non-hematopoietic peripheral blood stem cells; *Remestemcel-L* third-party/allogenic bone marrow of unrelated and human leukocyte antigen (HLA)-unmatched healthy adult donors; *NestCell®* MSC therapy produced by Cellavita; *SBI-101 therapy* extracorporeal mesenchymal stromal cell therapy; *SBI-101* a biologic/device combination product that combines two components: allogeneic human MSCs and an FDA-approved plasmapheresis device; *AT-MSCs* adipose tissue mesenchymal stem cells; *EV-MSCs* extracellular vesicles derived from MSCs; *WJUC-MSCs* Wharton’s jelly of umbilical cords mesenchymal stem cells; *UC-MSCs* umbilical cord mesenchymal stem cells; *CB-MSC* cord blood MSC; *AT-MSCs-Exo* exosomes derived from allogeneic AT-MSCs; *Om-MSCs* olfactory mucosa MSCs; *CM* conditioned media; *PLX-PAD* placental mesenchymal-like adherent stromal cells; *IV* intravenous; *IM* intramuscular; *N.A.* not applicable

**Table 4:** The investigated outcomes of the ongoing clinical trials using MSCs and MSC-derived exosomes to treat COVID-19 patients

Clinical trial identifier	Primary Outcome Measure	Secondary Outcome Measure
#NCT04348461	1. Efficacy of the administration assessed by survival rate [time frame, 28 days] 2. Safety of the administration by adverse event rate [time frame, 6 months].	N.A.
NCT04467047	1. Overall survival [time frame, 60 days] 2. Assessment of overall survival at 30 days post-intervention	Changes on inflammatory CRP, hospital stay, oxygenation index (PaO_2_/FiO_2_), evaluation of functional respiratory changes: PaO_2_/FiO_2_ ratio, Improvement in Liao’s score (2020), radiological improvement [time frame, 60 days], COVID19 PCR negativity [time frame, 28 days].
NCT04473170	Adverse reactions incidence, rate of mortality within 28-days, time to clinical improvement on a seven-category ordinal scale [time frame, day 0–28]	1. Assessment of the immune response profile. Immune response profile characterized according the biomarkers: CD3, CD4, CD8, CD11c, CD14, CD16, CD19, CD20, CD25, CD27, CD28, CD38, CD45, CD45RA, CD45RO, CD56, CD57, CD66b, CD123, CD127, CD161, CD294, CCR4, CCR6, CCR7, CXCR3, CXCR5, HLA-DR, IgD, and TCRγδ, for the identification of immune cells and subsets analysis; and the humoral Immune profile: IgG, IgA, IgM levels [time frame, Days 0, 14, and 28]. 2. Assessment of acute-phase serum markers. Complete Blood Counts (CBC), acute-phase proteins and Inflammatory markers: CRP, ESR, LDH, procalcitonin (PCT), ceruloplasmin, haptoglobin, alpha 1 antitrypsin, IL-6, ferritin C3, PT, fibrinogen and D-dimer [time frame, days 0, 14, and 28].
NCT04349540	Comparison of inflammatory/immunological biomarkers < 72 h after development of oxygen requirement [time frame, 72 h]	1. Overall survival at 30 and 100 days after development of oxygen requirement, those on immunosuppression. 2. Survival in SCT patients who are vs are not ongoing immunosuppression [time frame, days 30, and 100]. 3. Proportion of patients requiring mechanical ventilation [time frame, day 30]. 4. Incidence of secondary HLH (as defined by HS score) [time frame, day 30].
#ChiCTR2000029990	Improved respiratory system function (blood oxygen saturation) recovery time	N.A.
NCT04466098	Incidence of grade 3–5 infusional toxicities and predefined hemodynamic or respiratory adverse events related to the infusion of MSCs [time frame, within 6 h of the start of the infusion].	1. Incidence of a reduction in one or more biomarkers of inflammation by day 7 [time frame, day 7 after first infusion] 2. Trend changes in PaO_2_:FiO_2_ ratio, mean airway pressure, in peak pressure, plateau pressure, PEEP [time frame, on the day of screening and on days 3, 7 and 14 after first infusion]. 3. Incidence of mortality [time frame, 28 days after first infusion]. 4. Incidence of mortality [time frame, 100 days after first infusion]. 5. Number of ICU-free days [time frame, 28 days after first infusion] 6. Number of days alive and ventilator-free composite score 3 [time frame, 28 days after first infusion]. 7. Change in acute lung injury (ALI) score 2 [time frame, baseline and day 28 after first infusion]. 8. Incidence of serious adverse events [time frame, 28 days after first infusion] 9. Number of days alive off supplemental oxygen [time frame, 100 days after first infusion].
NCT04445220	Safety and tolerability as measured by incidence of IP-related serious adverse events [time frame, outcomes and serious adverse events through Day 180].	N.A.
NCT04447833	The incidence of TRAEIs [time frame, From drug administration to day 10 post-infusion]. TRAEIs: • → New ventricular tachycardia, ventricular fibrillation or asystole within 10 days after infusion • → New cardiac arrhythmia requiring cardioversion within 10 days after infusion • → Clinical scenario consistent with transfusion incompatibility or transfusion-related infection, thromboembolic events (e.g.. pulmonary embolism), cardiac arrest or death within 10 days after infusion	1. Safety; All-cause mortality [time frame, 60 days post-infusion, 6 months, 1, 2, 3, 4, and 5 years post-infusion]. 2. Changes in leucocytes [time frame, baseline (pre-infusion), day 1, 2, 3, 4, 7 and 10 post-infusion, 6 months, 1, 2, 3, 4, and 5 years post-infusion]. 3. Changes in Trombocytes [time frame, baseline (pre-infusion), day 1, 2, 3, 4, 7 and 10 post-infusion, 6 months, 1, 2, 3, 4, and 5 years post-infusion]. 4. Changes in plasma concentration of C-reactive protein (CRP) [time frame, baseline (pre-infusion), day 1, 2, 3, 4, 7 and 10 post-infusion, 6 months, 1, 2, 3, 4, and 5 years post-infusion]. 5. Changes in plasma concentration of prothrombin complex (PK) [time frame, baseline (pre-infusion), day 1, 2, 3, 4, 7 and 10 post-infusion, 6 months, 1, 2, 3, 4, and 5 years post-infusion]. 6. Changes in plasma concentration of Creatinine [time frame, baseline (pre-infusion), day 1, 2, 3, 4, 7 and 10 post-infusion, 6 months, 1, 2, 3, 4, and 5 years post-infusion]. 7. Changes in plasma concentration of Aspartate amino transferase (ASAT) [time frame, Baseline (pre-infusion), day 1, 2, 3, 4, 7 and 10 post-infusion, 6 months, 1, 2, 3, 4, and 5 years post-infusion]. 8. Changes in plasma concentration of Alanine amino transferase (ALAT) [time frame, Baseline (pre-infusion), day 1, 2, 3, 4, 7 and 10 post-infusion, 6 months, 1, 2, 3, 4, and 5 years post-infusion]. 9. Changes in plasma concentration of N-terminal pro-brain natriuretic peptide (NT-proBNP) [time frame, baseline (pre-infusion), day 1, 2, 3, 4, 7 and 10 post-infusion, 6 months, 1, 2, 3, 4, and 5 years post-infusion]. 10. Changes in blood pressure [time frame, baseline (pre-infusion), day 1, 2, 3, 4, 7 and 10 post-infusion, 6 months, 1, 2, 3, 4, and 5 years post-infusion]. 11. Changes in body temperature [time frame, baseline (pre-infusion), day 1, 2, 3, 4, 7 and 10 post-infusion, 6 months, 1, 2, 3, 4, and 5 years post-infusion]. 12. Efficacy; changes in pulmonary compliance [time frame, baseline (pre-infusion), day 1, 2, 3, 4, 7 and 10 post-infusion]. 13. Efficacy; changes in driving pressure (plateau pressure—PEEP) [time frame, baseline (pre-infusion), day 1, 2, 3, 4, 7 and 10 post-infusion]. 14. Efficacy; changes in oxygenation (PaO_2_/FiO_2_) [time frame, baseline (pre-infusion), day 1, 2, 3, 4, 7, and 10 post-infusion]. 15. Efficacy; duration of ventilator support [time frame, baseline (pre-infusion), day 1, 2, 3, 4, 7, 10 and 60 post-infusion]. 16. Efficacy; pulmonary bilateral infiltrates [time frame, baseline (pre-infusion), day 1, 2, 3, 4, 7 and 10 post-infusion, 6 months, 1, 2, 3, 4, and 5 years post-infusion]. 17. Efficacy; sequential organ failure assessment (SOFA) score [time frame, baseline (pre-infusion), day 1, 2, 3, 4, 7 and 10 post-infusion, end of ICU]. 18. Efficacy; hospital stay [time frame, day 60 post-infusion]. 19. Lung function [time frame, day 60 post-infusion, 6 months, 1, 2, 3, 4, and 5 years post-infusion]. 20. Lung fibrosis [time frame, baseline (pre-infusion), day 1, 3, 7 and 10 post-infusion, 6 months, 1, 2, 3, 4, and 5 years post-infusion]. 21. 6 min walk test [time frame, 6 months, 1, 2, 3, 4, and 5 years post-infusion]. 22. Changes in quality of life [time frame, 6 months, 1, 2, 3, 4, and 5 years post-infusion]. 23. Blood biomarkers [time frame, baseline (pre-infusion), day 1, 2, 3, 4, 7 and 10 post-infusion, 6 months, 1, 2, 3, 4, and 5 years post-infusion]. 24. Sensitization test [time frame, baseline (pre-infusion), day 60 post-infusion]. Sensitization tests (test for donor-specific antibodies) against KI-MSC-PL-205 donor.
NCT04457609	Clinical improvement: presence of dyspnea, presence of sputum, fever, ventilation status, blood pressure, heart rate, respiratory rate, oxygen saturation [time frame, 15 days].	Leukocyte, lymphocytes, CO_2_, HCO_3_, blood base excess level, blood oxygen partial pressure, O_2_ saturation, blood PH level, CRP, SGOT/SGPT (AST/ALT), ureum/creatinine, eGFR, sodium, potassium, chloride, procalcitonin, albumin, bilirubin, D-dimer level, fibrinogen, troponin, NT proBNP level [time frame, 15 days]. Measure leukemia inhibiting factor, IL-6, IL-10, ferritin, CXCR3, CD4, CD8, CD56 [time frame, 7 days]. Radiologic Improvement from chest X-ray/CT Scan [time frame, 15 days].
NCT04397471	Determine feasibility of recruiting healthy volunteers in a clinically useful timeframe. [time frame, 3 or more participants recruited in 1 month]. Manufacture a cell-based product suitable for clinical use [time frame, successfully opening the next phase of the trial in approx. 2 months].	Establishment of a robust process of production [time frame, successfully opening the next phase of the trial in approx. 2 months]. Production of stability data to be used in the MHRA dossier for the COMET clinical trial. [time frame, successfully opening the next phase of the trial in approx. 2 months] Production of cell-based products to be administered to COVID-19 patients with severe pneumonitis. [time frame, successfully opening the next phase of the trial in approx. 2 months]. Analysis of cells for understanding production, manufacture and related research. [time frame, Successfully opening the next phase of the trial in approx. 2 months].
NCT04461925	Changes of oxygenation index PaO_2_/FiO_2_, most conveniently the P/F ratio [time frame, up to 28 days]. Changes in length of hospital stay [time frame, up to 28 days]. Changes in mortality rate [time frame, up to 28 days].	Changes of С-reactive protein (CRP, mg/L) [time frame, At baseline, Day 1, Week 1, Week 2, Week 4, Week 8]. Evaluation of pneumonia improvement [time frame, at baseline, Day 1, Week 1, Week 2, Week 4, Week 8]. Duration of respiratory symptoms (difficulty breathing, dry cough, fever, etc.) [time frame, at baseline, day 1, week 1, week 2, week 4, week 8]. Peripheral blood count recovery time [time frame, at baseline, day 1, week 1, week 2, week 4, week 8].
NCT04428801	Tolerability and acute safety of cell infusion by assessment of the total number of AEs/SAEs related and non-related with the medication [time frame, 6 months]. The overall proportion of subjects who develop any AEs/SAEs related and non-related with the AdMSC infusions as compared to the control group [time frame, 6 months]. COVID-19 incidence rates in both the study and control groups [time frame, 6 months].	1. The proportion of subjects who are infected by SARS-Cov-2 measured by PCR or other nuclear level-based SARS-Cov-2 testing in respiratory tract specimens (oropharyngeal samples) collected by oropharyngeal swab using the CDC standard method. [time frame, 6 months]. 2. The proportion of subjects who are infected by SARS-Cov-2 virus develop symptoms including mild, classic, severe and critical sever cases between study group and control group. [time frame, 6 months]. 3. Change of proportion of subjects who are infected by SARS-Cov-2 and develop IgM/IgG antibodies against SARS-Cov-2 between study group and control group. [time frame, 6 months]. 4. Change of lymphocyte count in white blood cell counts, PaO_2_ arterial blood gas from the baseline [time frame, 6 months]. 5. Compare the proportion of subjects who develop severe COVID-19 pneumonia cases, mortality rates, C-reactive protein (CRP), D-dimer (mg/L), procalcitonin (μg)/L, pro-type B natriuretic peptide (pro-BNP) (pg/mL), bilirubin, creatinine for both study and control groups [time frame, 6 months]. 6. Change in blood test values for cytokine panels (IL-1β, IL-6, IL-8, IL-10, TNFα) from the baseline [time frame, 6 months]. 7. Change in blood test values for cytokine panels (IL-1β, IL-6, IL-8, IL-10, TNFα) from the baseline [time frame, 6 months] 8. The proportion of subjects from SARS-CoV-2 RT-PCR positive to negativity in respiratory tract specimens (oropharyngeal samples) collected by oropharyngeal swab using the CDC standard method. as compared to control group [time frame, 6 months]. 9. Quantifying viral RNA in stool for baseline and final follow-up. [time frame, 6 months].
NCT04416139	Functional Respiratory changes: PaO_2_/FiO_2_ ratio, Changes in body temperature, cardiac changes: Heart rate per minute, respiratory rate [time frame, 3 weeks].	General biochemical changes in leukocytes, lymphocytes, platelets, fibrinogen, pocalcitonin, ferritin, D-dimer, C-reactive protein, Inflammatory cytokine TNFa, IL10, IL1, IL6, IL 17, VEGF, radiological changes (CT), immunological changes on T cell, dendritic cells, CD4+ T, CD8+ T, NK cell, RNA detection by SARS-Cov2 PCR, and adverse events [time frame, 3 weeks].
NCT04429763	Clinical deterioration or death [time frame, 4 weeks].	N.A.
NCT04444271	Overall survival [time frame, 30 days post-intervention].	1. Clinical improvement [time frame, 30 days]. 2. Time of COVID19 PCR negativity [time frame, day 1, 3, 7, 10, 14]. 3. Radiological improvement (day 15 and day 30 assessment) [time frame, day 15 and day30]. 4. Days required to discharge from hospital [time frame, 30 days post-admission].
NCT04456361	Oxygen saturation [time frame, baseline, and at days 2, 4, and 14 post-treatment].	Oxygen pressure in inspiration, ground-glass opacity, pneumonia infiltration, LDH, CRP, D-dimer ferritin [time frame, Baseline, and at days 4 and 14 post-treatment].
NCT04366271	Mortality due to lung involvement due to SARS-CoV-2 infection at 28 days of treatment [time frame, 28 days].	1. Mortality due to lung involvement due to SARS-CoV-2 infection at 14 days of treatment [time frame, 14 days]. 2. Mortality from any cause at 28 days [time frame, 28 days]. 3. Days without mechanical respirator and without vasopressor treatment for 28 days [time frame, 28 days]. 4. Patients alive without mechanical ventilation and without vasopressors on day 28 [time frame, 28 days]. 5. Patients alive and without mechanical ventilation on day 14 [time frame, 14 days]. 6. Patients alive and without mechanical ventilation on day 28 [time frame, 28 days]. 7. Patients alive and without vasopressors on day 28 [time frame, 28 days]. 8. Days without vasopressors for 28 days [time frame, 28 days]. 9. Patients cured at 15 days [time frame, 15 days]. 10. Incidence of treatment-emergent adverse events [time frame, 1 year].
NCT04371393	Number of all-cause mortality [time frame, 30 days].	1. Number of days alive off mechanical ventilatory support [time frame, 60 days]. 2. Number of adverse events [time frame, 30 days]. 3. Number of participants alive at day 7, 14, 60, 90. 4. Number of participants with resolution and/or improvement of ARDS on days 7, 14, 21, and 30. 5. Change from baseline of the severity of ARDS on days 7, 14, 21, and 30. 6. Length of stay [time frame, 12 months] 7. Clinical improvement scale on days 7, 14, 21 and 30; change CRP concentration on days 7, 14, 21, and 30. 8. Change in IL-6 and IL-8 inflammatory marker level on days 7, 14, 21 and 30; change in TNF-alpha inflammatory marker level on days 7, 14, 21, and 30.
NCT04313322	Clinical outcome, CT Scan, RT-PCR results [time frame, 3 weeks].	RT-PCR results [time frame, 8 weeks].
NCT04452097	1 Incidence of infusion-related adverse events [time frame, day 3]. 2 Incidence of any treatment-emergent adverse events (TEAEs) and treatment-emergent serious adverse events (TESAEs) [time frame, day 28].	Selection of an appropriate dose of the hUC-MSC product for the following phase 2 study [time frame, Day 28].
NCT04315987	Change in clinical condition [time frame, 10 days].	1. Rate of mortality, respiratory rate, hypoxia, PaO_2_/FiO_2_ ratio, changes of blood oxygen, side effects [time frame, 10 days]. 2. CD4+ and CD8+ T cell count [time frame, days 1, 2, 4, 6 and 8]. 3. Complete blood count, cardiac, hepatic, and renal profiles; [time frame, days 1, 2, 4, 6, and 8].
NCT04252118 (preliminary for NCT04288102)	Size of lesion area by chest radiograph or CT [time frame, at baseline, day 3, 6, 10, 14, 21, 28] Side effects in the MSCs treatment group [time frame, at baseline, day 3, 6, 10, 14, 21, 28, 90 and 180].	1. Improvement of clinical symptoms including duration of fever and respiratory [time frame, at baseline, day 3, 6, 10, 14, 21, 28]. 2. Time of nucleic acid turning negative, CD4+ and CD8+ T cell count, alanine aminotransferase, C-reactive protein, creatine kinase [time frame, at baseline, day 3, 6, 10, 14, 21, 28, 90 and 180]. 3. Rate of mortality within 28-days [time frame, day 28].
NCT04288102	Change in lesion proportion (%) of full lung volume from baseline to day 28. [time frame, day 28].	1. Change in lesion proportion (%) of full lung volume from baseline to day 10 and 90 [time frame, day 10, day 90]. 2. Change in consolidation lesion proportion (%) of full lung volume from baseline to day 10, 28 and 90. [time frame, day 10, 28, and 90]. 3. Change in ground-glass lesion proportion (%) of full lung volume from baseline to day 10, 28 and 90. [time frame, day 10, 28, and 90]. 4. Pulmonary fibrosis-related morphological features in CT scan at day 90. 5. Lung densitometry [time frame, days 10, 28, and 90]. 6. Lung densitometry: volumes histogram of lung density distribution (< − 750, − 750 to about − 300, − 300 to about 50, > 50) at day 10, 28 and 90. [time frame, days 10, 28, and 90]. 7. Time to clinical improvement in 28 days. [time frame, day 28]. 8. Oxygenation index (PaO_2_/FiO_2_) [time frame, days 6, 10, and 28] 9. Duration of oxygen therapy (days) [time frame, day 28 and 90]. 10. Blood oxygen saturation [time frame, days 6, 10, and 28] 11. 6-min walk test [time frame, days 28 and 90]. 12. Maximum vital capacity (VCmax) [time frame, baseline, days 10, 14, 21, 28, and 90]. 13. Diffusing capacity (DLCO) [time frame, baseline, days 10, 14, 21, 28, and 90]. 14. mMRC (Modified Medical Research Council) dyspnea scale [time frame, day 28, Day 90]. 15. Changes of absolute lymphocyte counts and subsets, changes of cytokine/chemokine from baseline to day 6, 10, 28 and 90. [time frame, days 6, 10, 28, and 90]. 16. Adverse events, serious adverse events, all-cause mortality [time frame, day 0 through Day 90].
NCT04302519	Improvement time of ground-glass shadow in the lungs [time frame, 14 days].	1. Absorption of lung shadow absorption by CT scan-chest [time frame, 7, 14, 28 and 360 days] 2. Changes of blood oxygen [time frame, 3, 7 and 14 days]
NCT04273646	Pneumonia severity index [time frame, from baseline (0 w) to 12 week after treatment]. Oxygenation index (PaO_2_/FiO_2_) [time frame, from baseline (0 w) to 12 week after treatment].	1. Side effects [time frame, From Baseline (0 W) to 96 week after treatment]. 2. Survival, sequential organ failure assessment [time frame, day 28]. 3. C-reactive protein, procalcitonin, lymphocyte count, CD3+, CD4+ and CD8+ T cell count, CD4+/CD8+ratio [time frame, from baseline (0 W) to 12 week after treatment].
NCT04299152		1. Percentage of activated T cells, percentage of Th17 after therapy by flow cytometry [time frame, 4 weeks]. 2. Chest imaging changes by computed tomography (CT) scan of the chest, quantification of the SARS-CoV-2 viral load by real time RT-PCR [time frame, 4 weeks].
NCT04269525	Oxygenation index [time frame, on the day 14 after enrollment].	1. 28 day mortality rate. 2. Hospital stay [time frame, up to 6 months] 3. COVID-19 antibody test on the day 7, 14, and 28. 4. Improvement of lung imaging examinations on the day 7, 14, 28 5. White blood cell count, procalcitonin, lymphocyte count, IL-2, IL-4, IL-6, IL-10, TNF-α, γ-IFN, CRP, CD4+, CD8+, NK cells [time frame, on the day 7, 14, 28 after enrollment]
NCT04333368	Respiratory efficacy evaluated by the increase in PaO_2_/FiO_2_ ratio from baseline to day 7 in the experimental group compared with the placebo group [time frame, From baseline to day 7].	1. Lung injury score, oxygenation index, in-hospital mortality, mortality, ventilator-free days, number of days between randomization and the first day the patient meets weaning criteria meets PaO_2_/FiO_2_ > 200 (out of a prone positioning session) [time frame, from baseline to day 28]. 2. Cumulative use of sedatives, duration of use of sedatives, duration of use of neuromuscular blocking agents (other than used for intubation), use of neuromuscular blocking agents (other than used for intubation), ICU-acquired weakness and delirium, treatment-induced toxicity rate and adverse events up to day 28. 3. Quality of life at 1 year (EQ. 5D-3L quality of life questionnaire) [time frame, At 6 months and 12 months]. 4. Measurements of plasmatic cytokines (IL1, IL6, IL8, TNF-alpha, IL10, TGF-beta, sRAGE, Ang2) level [time frame, At day 1, 3, 5, 7, and 14]. 5. Anti-HLA antibodies plasmatic dosage [time frame, from baseline to day 14, and at 6 months].
NCT04276987	Adverse reaction (AE) and severe adverse reaction (SAE) Time to clinical improvement (TTIC) [time frame, up to 28 days].	Number of patients weaning from mechanical ventilation, duration (days) of ICU monitoring, vasoactive agents usage, mechanical ventilation supply, number of patients with improved organ failure and mortality rate within 28 days.
NCT04336254	Time to clinical improvement [time frame, 1–28 days].	Lung lesion, immune function (Th1 cytokines: IL-1β, IL-2, TNF-a, ITN-γ; Th2 cytokines: IL-4, IL-6, IL-10; immunoglobulins: IgA, IgG, IgM, and total IgE; Lymphocyte counts: CD3+, CD4+, CD8+, CD16+,CD19+, CD56+), time of SARS-CoV-2 clearance, blood test, SPO_2_, RR, body temperature, side effects in the treatment group, CRP [time frame, 1–28 days].
NCT04348435	Incidence of hospitalization for COVID-19, incidence of symptoms associated with COVID-19 [time frame, week 0 through week 26 (end of study)].	1. Absence of upper/lower respiratory infection [time frame, week 0 through week 26]. 2. Leukocyte differential, CRP, TNF alpha, IL-6, IL-10, glucose, calcium, albumin, total protein, sodium, total carbon dioxide, complete blood count (CBC) and complete metabolic profile (CMP) [time frame, weeks 0, 6, 14, 26].
NCT04352803	Incidence of unexpected adverse events, frequency of progression to mechanical ventilation, changes in length of mechanical ventilation, changes in length of weaning of mechanical ventilation, changes in length of hospital stay, changes in mortality rate [time frame, up to 28 days].	N.A.
NCT04366323	Safety of the administration assessed by adverse event rate [time frame, 12 months] Efficacy of the administration by survival rate [time frame, 28 days]	N.A.
NCT04349631	Incidence of hospitalization for COVID-19 [time frame, week 0 through week 26 (end of study)] Incidence of symptoms for COVID-19 [time frame, week 0 through week 26 (end of study)].	Absence of upper/lower respiratory infection [time frame, weeks 0 through 26] CBC, CMP, and IL-10, 6, TNF-alpha [time frame, Weeks 0, 6, 14, 26].
NCT04346368	Changes of oxygenation index (PaO_2_/FiO_2_) [time frame, at baseline, 6 h, day 1, 3, week 1, week 2, week 4, month 6] Side effects in the BM-MSCs treatment group [time frame, baseline through 6 months].	Clinical outcome, hospital stay, CT scan, changes in viral load, changes of CD4+, CD8+ cells count and concentration of cytokines, rate of mortality within 28-days, changes of C-reactive protein [time frame, From baseline to day 28].
NCT04382547	Number of cured patients [time frame, 3 weeks]	Number of patients with treatment-related adverse events [time frame, 3 weeks].
NCT04366063	Adverse events assessment [time frame, from baseline to day 28]. Blood oxygen saturation [time frame, from baseline to day 14].	1. Intensive care unit-free days [time frame, up to day 8]. 2. Clinical symptoms [time frame, from baseline to day 14]. 3. Respiratory efficacy [time frame, from baseline to day 7]. 4. Biomarkers concentrations in plasma [time frame, at baseline, 7, 14, 28 days after the first intervention].
NCT04437823	Safety and efficacy assessment of infusion associated adverse events [time frame, day 01 to day 30] Chest radiograph or chest CT scan [time frame, day 01 to day 30].	COVID-19 Quantitative real time PCR, Sequential Organ Failure Assessment (SOFA) score, evaluation of organ function (each organ system is assigned a value for 0 (normal) to 4 (highest degree of dysfunction)), rate of mortality, clinical respiratory changes [time frame, day 01 to day 30].
NCT04339660	The immune function (TNF-α, IL-1β, IL-6, TGF-β, IL-8, PCT, CRP) [time frame, 4 weeks]. Blood oxygen saturation [time frame, 4 weeks].	Rate of mortality within 28-days, size of lesion area by chest imaging, CD4+ and CD8+ T cells count, peripheral blood count recovery time, duration of respiratory symptoms (fever, dry cough, difficulty breathing), COVID-19 nucleic acid negative time [time frame, at baseline, day 1, 2, 7, week 2, week 3, week 4].
NCT04392778	Clinical improvement [time frame, 3 months].	Lung damage improvement, SARS-Cov-2 viral infection laboratory test, blood test [time frame, 3 months].
NCT04371601	Changes of oxygenation index (PaO_2_/FiO_2_), blood gas test [time frame, 12 months].	Detection of TNF-α levels, IL-10 levels, immune cells that secret cytokines, including CXCR3+, CD4+, CD8+, NK+ cells, and regulatory T cells (CD4+ CD25+ FOXP3+ Treg cells). Changes of oxygenation index (PaO_2_/FiO_2_), blood gas test, changes of c-reactive protein and calcitonin [time frame, 1, 3, 6, 12 months].
NCT04355728	Incidence of pre-specified infusion associated adverse events [time frame, day 5]. Incidence of severe adverse events [time frame, 90 days].	1. Survival rate after 90 days post first infusion [time frame, 90 days]. 2. Small Identification Test (SIT) scores [time frame, At baseline, day 18 and day 28]. 3. CBC, CMP, D-dimer, and alloantibodies levels [time frame, Baseline, 28 days].
NCT04362189	Interleukin-6, C reactive protein, oxygenation, TNF alpha, IL-10 [time frame, day 0, 7. 10]. Return to room air (RTRA) [time frame, day 0, 3, 7, 10, 28].	1. CBC, CMP, D-dimer, INR, CD4+/CD8+ ratio, NK cells [time frame, screening, day 0, 7, 10]. 2. CT scan [time frame, days 0 and 28]. 3. PCR test for SARS-CoV-2 [time frame, day 0, 3, 7, 10].
NCT04390152	Intergroup mortality difference with treatment [time frame, 28 days].	1. Number of patients with treatment-related adverse events [time frame, 6 months]. 2. Difference in days of mechanical ventilation between groups [time frame, From ICU admission to 180 days]. 3. Median reduction of days of hospitalization, reduction of days of oxygen needs [time frame, from hospital admission to 180 days]. 4. Difference in APACHE II score between groups [time frame, baseline and 7 days] 5. CBC, CMP, LDH, IL-6, IL-10, TNF-alpha [time frame, baseline to 7 days].
NCT04377334	Lung injury score [time frame, day 10].	D-dimer, immune cell phenotype, pro-resolving lipid mediators, cytokines, chemokines day 0, 1, 2, 3, 10 and 15, survival (day 10 and 28), extubation (day 28), lymphocyte subpopulations, SARS-CoV-2-specific antibody titers and complement molecules (C5-C9) (day 0, 5 and 10).
NCT04331613	Adverse reaction (AE) and severe adverse reaction (SAE), changes of lung imaging examinations [time frame, Within 28 days after treatment].	CBC, CMP, IL-1beta, IL-2, IL-6, IL-8, lactate, procalcitonin, CRP, CK, and rate of all-cause mortality within 28 days.
NCT04390139	All-cause mortality at day 28 [time frame, day 28].	Safety, need for treatment with rescue medication, duration of mechanical ventilation, ventilator-free days, evolution of PaO_2_/FiO_2_ ratio, SOFA index, APACHE II score, duration of hospitalization, evolution of markers of immune response (leucocyte count, neutrophils), feasibility of MSC administration, LDH, ferritin.
NCT04400032	Treatment-related adverse events [time frame, at time of infusion-12 months].	Number of participants alive and number of participants with ventilator-free by day 28.
NCT04398303	Mortality at day 30 [time frame, 30 days post-treatment].	Improvement in ventilator settings [time frame, 28–30 days post-treatment].
NCT04365101	Phase 1: frequency and severity of adverse events (AE), rate of clearance of SARS-CoV-2, clinical improvement [time frame, up to 12 months]. Phase 2: Time to clearance of SARS-CoV-2, clinical improvement by NEWS2 Score [time frame, up to 28 days].	Mortality rate and impact on sequential organ failure assessment (SOFA) score pulmonary clearance [time frame, up to 28 days].
NCT04393415	Clinical improvement [time frame, 2 weeks].	N.A.
NCT04397796	Adverse event and mortality rates [time frame, 30 days] Ventilator-free days [time frame, 60 days].	1. Change in NEWS from baseline (NEWS of ≤ 2 [time frame, 30 days]). 2. SOFA score on days 8, 15, 22, and 29.
NCT03042143	Oxygenation index (OI) [time frame, day 7]. Incidence of serious adverse events (SAEs) [time frame, 28 days].	1. SOFA score [time frame, days 4, 7 and 14]. 2. Respiratory compliance (Crs) [time frame, days 4, 7, and 14]. 3. Oxygenation index [time frame, days 4, and 14]. 4. Ventilation and pulmonar function [time frame, 28 and 90 days].
NCT04345601	Treatment-related serious adverse events [time frame, 28 days post cell infusion]. Change in clinical status at day 14 [time frame, 14 days post cell infusion].	N.A.
NCT04361942	Proportion of patients who have achieved withdrawal of invasive mechanical ventilation [time frame, 0–7 days]. Mortality rate [time frame, 28 days].	Proportion of patients who have achieved clinical response (0–7 days) and radiological responses (0–28 days).
NCT04333368	Respiratory efficacy evaluated by the increase in PaO_2_/FiO_2_ [time frame, from baseline to day 7].	1. Lung injury score, Oxygenation index, In-hospital mortality, mortality, ventilator-free days, proportion of PaO_2_/FiO_2_ > 200, cumulative use and duration of sedatives and neuromuscular blocking agents, ICU-acquired weakness and delirium, treatment-induced toxicity rate and adverse events up to day 28. 2. Quality of life at one year (EQ. 5D-3L quality of life questionnaire) [time frame, at 6 months and 12 months] 3. Measurements of plasmatic cytokines (IL1, IL6, IL8, TNF-alpha, IL10, TGF-beta, sRAGE, Ang2) level [time frame, At day 1, 3, 5, 7 and 14]. 4. Anti-HLA antibodies plasmatic dosage [time frame, from baseline to day 14, and at 6 months].
NCT04389450	Number of ventilator-free days [time frame, 28 days].	1. All-cause mortality [time frame, 28 days] 2. Duration of mechanical ventilation [time frame, 8 weeks].
NCT04367077	Ventilator-free days, safety and tolerability as measured by the incidence of treatment-emergent adverse events [time frame, day 0–28].	1. All-cause mortality [time frame, Day 60] 2. Ranked hierarchical composite outcome of alive and ventilator-free [time frame, Day 28]. 3. Ventilator-free days [time frame, day 0–60].

*PEEP* positive end-expiratory airway pressure, *AEs/SAEs* adverse events and severe adverse events, *CRP* C-reactive protein, *LDH* lactate dehydrogenase. APACHE II is a prognostic score based on 12 different items obtained in the first 24 h of ICU admission. It ranges from 0 to 71 points. A higher score is associated with higher mortality. *TRAEIs*, Pre-specified treatment-related adverse events of interest; *NEWS2*, National Early Warning Score 2 Score; *NEWS*: respiration rate, oxygen saturation, any supplemental oxygen, temperature, systolic blood pressure, heart rate, level of consciousness; *SOFA*, respiration, coagulation, liver, cardiovascular, central nervous system, and renal

Another recently published study has tested the safety and efficacy of allogeneic AT-MSCs in 13 COVID-19 adult patients under invasive mechanical ventilation [169]. The patients had received previous anti-inflammatory and/or antiviral drugs, including lopinavir/ritonavir, steroids, tocilizumab and/or hydroxychloroquine, among others. Thirteen patients have been enrolled in the trial and have received two IV doses of AT-MSC 0.98 × 10^6^ per kg of body weight, 3 days apart. The treatment has been followed by a reduction in inflammatory parameters (CRP, ferritin, LDH, IL-6) as well as increase in B-lymphocytes (67%) and CD4^+^ and CD8^+^ (100%) T lymphocytes). Remarkably, a reduction of D-dimer and fibrinogen 5 days after the first dose of AT-MSCs has been observed in most patients, and the patients do not develop a thromboembolic event [169, 170].

Furthermore, a recent clinical trial has been employed using MSC-derived exosomes. A prospective nonrandomized open-label cohort study by Sengupta et al. has evaluated the safety and efficacy of exosomes (ExoFlo™) obtained from allogeneic BM-MSCs as treatment for severe COVID-19. A total of 24 patients have received 15 ml of ExoFlo™. The treatment resulted in significant improvement in absolute neutrophil count and lymphopenia, with a decline in CRP, ferritin, and D-dimer. To our knowledge, this is the first published clinical study to use IV administration of BM-MSC-derived exosomes as treatment for COVID-19 [154]. These findings indicate that MSCs and their exosomes are promising options for treating ARDS associated with respiratory viral infections.

## Points to consider in designing MSC clinical trials for COVID-19

Only regulated and compliant clinical trials can demonstrate and provide mechanistic and translational insights on the role of MSCs in ALI and ARDS in COVID-19. Designing randomized controlled trials (RCT) with setting clear inclusion and exclusion criteria will aid in laying the foundation for a safe and effective stem cell-based therapy in COVID19. Carrying a multicenter RCT (MRCT) versus a single center study is a decision that could further enhance advancing phases of previously successful phase 1/2 clinical trials. MRCT allows for capturing adequate sample size to reach significance and eliminates selection bias and confounding factors [171]. However, one has to define a consensus on cell-characterization, inclusion/exclusion criteria, and outcome measures. Although there is no consensus on the criteria of MSCs clinical trials in COVID-19 as of yet, the most common inclusion criteria are confirmed SARS-COV-2 by RT-PCR from respiratory sample, respiratory failure requiring intubation and ventilator, and meeting criteria of ARDS (PaO_2_/FiO_2_ ratio < 200 mmHg). Among the most common exclusion criteria are other causes of ARDS not attributed to COVID-19, negative RT-PCR for SARS-COV-2, pregnancy, recent history of thromboembolism, active malignancy, or previous immunosuppressive treatment. Primary outcome measures should aim at identifying safety and efficacy, by measuring adverse event rate and survival rate, respectively. Furthermore, setting a clear time frame for capturing the primary outcomes should be identified. Secondary outcome measures are also important in assessing the success of MSCs/MSC-EVs in COVID-19, mainly looking at long-term effects and measured therapeutic input. Secondary outcomes measures could include validated clinical assessment scoring like the sequential organ failure assessment (SOFA) which looks at multiple organ system functions (respiration, coagulation, liver, cardiovascular, central nervous system, and renal). Specifically, secondary outcome measure should focus on pulmonary function (PaO_2_/FiO_2_ > 200); therefore, setting a clear lung injury assessment score at baseline and at defined time frames following therapy. Finally, to be able to provide mechanistic and translational insights on the role of MSCs in ALI and ARDS in COVID-19, obtaining a basic complete blood count (CBC), comprehensive metabolic panel (CMP), and key inflammatory markers, as well as measurement of key cytokines (IL1, IL6, IL8, TNF-alpha, IL10, TGF-beta, sRAGE, Ang-2) at baseline and at defined time frames following therapy is crucial.

One of the key points in designing the clinical trial is the MSC dose and route of administration. IV route is the most commonly used method for systemic delivery of MSCs in the majority of clinical trials, with much fewer trials using intra-arterial (IA) injection [172]. The most frequently utilized route of MSC administration in ARDS and COVID-19 is also via IV infusion. Of interest, IV route remains the most well studied route used for MSC delivery in pulmonary diseases [172]. Regardless of the site of inflammation and tissue injury, and opposing to the old concept that MSCs only migrate to the site of injury following IV administration, MSCs are mostly trapped in lungs and undergo phagocytosis within 24 h [173]. In the majority of clinical indications, human MSCs are frequently transfused IV at doses ranging from 1 to 2 million cells/kg and never exceeding a dose of 12 million cells/kg [172, 174]. The median dose for IV route is 1 × 10^8^ MSCs per patient per total dose. Analysis of MSC trials using IV route indicated minimal effective doses (MEDs). Efficacy dose-response outcome data indicated a narrower MED range of MSCs ranging from 100 to 150 million, where either higher or lower has been less efficient [172].

## Challenges in treating COVID-19 using MSCs and their exosomes

One of the most significant challenges for MSC therapies is to optimize MSC homing efficiency. IV administration of MSCs shows low homing efficiency where cells get trapped in the pulmonary capillaries [175], a process that has been partially explained by insufficient production of homing factors, such as CXCR4, on MSCs [176, 177]. It has been reported that the in vitro propagation of MSCs gradually leads to dramatic reduction in the expression of homing factors [178, 179]. Several strategies have been used to improve MSC homing capacity, including targeted administration, genetic modification, magnetic guidance, in vitro priming, cell surface modification, and radiotherapeutic techniques [180, 181].

Further studies are needed to define the optimal source and dose of MSCs, administration route, the time window of MSC administration, and dose frequency (single vs. multiple-dose regimen). Due to MSC expression of tissue factor (TF/CD142), which triggers the coagulation, a pro-coagulation can be triggered ultimately leading to thromboembolic events following infusion. Thus, the use of anti-coagulant during MSC administration can be considered during administration guidelines and protocols [182]. Due to the trapping effect of MSCs occurring in the lung, a justification for the high doses of MSCs has been proposed [183]. Therefore, MSCs could be genetically modified to overexpress selected genes in order to increase in their engraftment. Pre-treatment with a series of preconditioning approaches could also promote MSCs therapeutic effects and enhance their survival in the lung. Of note, challenges with autologous MSC transplantation in ARDS are demonstrated by their impaired potential due to the immunomodulatory effects of bone marrow MSCs [184].

As extracorporeal membrane oxygenation (ECMO) is among the salvage therapy for refractory respiratory failure in the context of acute respiratory compromise associated with SARS-CoV-2, it is critical to learn how MSCs would act in such setting [185]. IV administration of MSCs has been found to attach to membrane oxygenator fibers during ECMO in an in vitro ARDS model, leading to a significant decrease in the flow through the circuit [186]. Intratracheal and IV infusions of MSCs before ECMO or during a pause in the flow are some suggested strategies to overcome this limitation. Interestingly, a more recent study demonstrated an enhanced endogenous MSC mobilization in patients with ARDS undergoing ECMO [187]. Therefore, intratracheal administration of MSCs might be an option in ARDS requiring continuous high-flow ECMO.

The fate of MSCs after infusion also remains to be investigated. Cell migration and distribution studies have shown that the majority of MSCs localize to the lungs after IV infusion [188, 189]. Intravascular arrest of MSCs is due to MSC’s diameter ranging from 10 to 20 μm, bigger than the width of the pulmonary micro-capillaries [190]. However, following IV administration, MSCs still tend to migrate to sites of injury and move from the lungs to other organs, such as the liver and spleen [191, 192]. Nevertheless, challenges remain in interpreting this data as this tracking could be detecting phagocytosed MSCs. This is in line with the evidence showed that most of MSCs become apoptotic after administration [193]. To avoid poor cell survival following MSC transplantation, several preconditioning strategies have been proposed. Ang1-preconditioned cell survival was significantly increased via increased Akt phosphorylation. This has further reduced the apoptotic rate in vitro via increased expression of B cell lymphoma protein 2 (Bcl-2) and the ratio of Bcl-2/Bcl-associated X (Bax) [193]. Several priming strategies with pharmacological agents, inflammatory cytokines or mediators, hypoxia, and biomaterial have been shown to enhance the therapeutic efficacy of MSC transplantation. MSCs enhanced trafficking and homing to sites of injury is demonstrated in the high expression of chemokine receptors, such as CXCR4, CXCR7, and CX3CR1 [194]. Finally, although several in vitro culturing strategies have been developed to mimic the natural MSC niche, preserving the function and quality of a scalable clinical-grade cell expansion remain a challenge.

The adult sources of MSCs include painful and invasive procedures with possible donor site morbidity [195]. Regardless the origin, the MSCs display heterogeneity in their abilities to propagate and differentiate [196]*.* The differences in the properties of MSCs are associated with the variations in the age of the donor, method of MSC isolation, and in vitro culturing approaches. As an alternative source, hPSCs could differentiate into unlimited number of MSCs, displaying MSC characteristics [55, 197, 198]. Further studies are needed to extensively examine the differences between MSCs isolated from different tissues and those derived from hPSCs.

A systemic procoagulant state has been observed in severely ill COVID-19 patients, which tends to result in poor outcome. Such patients are at high risk of disseminated intravascular coagulation (DIC) and thromboembolism. Looking at the risk of hypercoaguable state in COVID-19 patients, the safety profile of MSCs could be further challenged [199]. DIC and thromboembolism taking place after the administration of TF/CD142-expressing MSC products have been reported [182]. The production of the highly procoagulant tissue factor TF/CD142 between products could vary. BM-MSCs have the lowest TF/CD142 expression, whereas ASC display the highest expression profile [182]. Finally, the dynamics of the current pandemic and the rising global demand highlight the need for scalable manufacturing required to provide enough doses of MSC product of high quality in a reproducible and timely manner.

## Conclusion and future perspectives

MSCs have a potential therapeutic function in COVID-19, which is displayed in their ability to enhance alveolar fluid clearance and promote epithelial and endothelial recovery through transfer of EV components together with the cell-cell contact as well as their secreted soluble factors. As ACE2 is widely expressed in other tissue types in addition to lungs, it is intuitive to consider MSC effect on the other organs as well. MSC treatment may reduce the progression of ARDS in severely ill COVID-19 patients with multiple organ failure.

Although several clinical trials have been recently registered to examine the safety and efficacy of MSCs as an emerging therapeutic option for COVID-19-induced disease, fewer studies have been published. Learning from these clinical trials, MSCs could exert its immunomodulatory and regenerative capacity in COVID-19 patients. Although there is no approved treatment for COVID-19 as of yet, MSC therapies continue to show improvement in the treatment of some of the leading causes of mortality in COVID-19 patients, namely acute ARDS, pneumonia, inflammation, and sepsis. The majority of these clinical trials are based on IV infusion of MSCs and their derived exosomes. Despite the clinical improvement witnessed, the safest and most effective route of MSC delivery into COVID-19 patients remains unclear, especially in the context of the heterogeneity of MSC-based products, intravascular arrest, and poor cell survival. IV infusions of poorly characterized MSC products remain one of the most significant drawbacks of MSC cell-based therapy, which could theoretically promote the risk for thromboembolism. The best delivery route for MSCs giving the highest positive effects with minimum toxic effects remains to be resolved. Whether any difference between IV, intratracheal, and intraperitoneal administration routes exists also remains unclear. Furthermore, elucidating the molecular mechanisms of MSCs during lung injury is crucial to understand their role in ARDS. The premature marketing of unproven stem cell therapy to the public resulted in the unfortunate increase of unregulated stem cell clinics; therefore, we cannot recommend any MSC-based treatment, which does not use characterized cell product, perform functional mechanisms, define variability in donor and tissue source, measure intermediate parameters, and define final patient endpoints(s), key steps that are common practice in FDA registered trials. Lack of consensus underlines major challenges to the clinical translation of MSC-based therapy.

Future studies should focus on developing genetically modified MSCs, generating significantly large number of EVs that could safely transfer different potent and effective therapeutic factors [114]. Finally, optimizing the clinical-grade production of MSCs as well as establishing a consensus on registered clinical trials based on cell-product characterization and mode of delivery would aid in laying the foundation for a safe and effective MSC-based therapy in COVID19.

## Data Availability

Not applicable.

## Abbreviations

ALI: Acute lung injury
ARDS: Acute respiratory distress syndrome
BM-MSCs: Bone marrow MSCs
CCL: CC chemokine ligand
COVID-19: 2019 novel coronavirus disease
CXCL: Chemoline (C-X-X motif) ligand
DCs: Dendritic cells
EVs: Extracellular vesicles
FGF-10: Fibroblast growth factor-10
GM-CSF: Granulocyte-macrophage colony stimulating factor
GVHD: Graft-versus-host-disease
HGF: Hepatocyte growth factor
HLA-GS: Human leukocyte antigen-G5
HO-1: Heme oxygenase-1
ICU: Intensive care unit
IDO: Indoleamine 2,3-dioxygenase
IGF1: Insulin growth factor 1
hPSCs: Human pluripotent stem cells
IL-1Ra: Interleukin-1 receptor antagonist
KGF-2: Keratinocyte growth factor-2
LIF: Leukemia inhibitory factor
MHC: Major histocompatibility complex
MERS: Middle Eastern respiratory syndrome
MSCs: Mesenchymal stem cells
MVs: Microvesicles
NO: Nitric oxide
PDL1: Programmed death-1
PGE2: Prostaglandin E2
SARS-CoV-2: Severe acute respiratory syndrome coronavirus
TGF-β: Transforming growth factor beta
TLRs: Toll-like receptors
VEGF: Vascular endothelial growth factor
WHO: World Health Organization
